# Molecular characterization of Thy1 expressing fear-inhibiting neurons within the basolateral amygdala

**DOI:** 10.1038/ncomms13149

**Published:** 2016-10-21

**Authors:** Kenneth M. McCullough, Dennis Choi, Jidong Guo, Kelsey Zimmerman, Jordan Walton, Donald G. Rainnie, Kerry J. Ressler

**Affiliations:** 1 Behavioral Neuroscience, Department of Psychiatry and Behavioral Sciences, Emory University, Atlanta, Georgia 30322, USA; 2 Division of Depression & Anxiety Disorders, McLean Hospital, Belmont, Massachusetts 02478, USA; 3 Department of Psychiatry, Harvard Medical School, Boston, Massachusetts 02478, USA; 4 School of Psychology, University of New South Wales, Sydney, New South Wales 2052, Australia

## Abstract

Molecular characterization of neuron populations, particularly those controlling threat responses, is essential for understanding the cellular basis of behaviour and identifying pharmacological agents acting selectively on fear-controlling circuitry. Here we demonstrate a comprehensive workflow for identification of pharmacologically tractable markers of behaviourally characterized cell populations. Thy1-eNpHR-, Thy1-Cre- and Thy1-eYFP-labelled neurons of the BLA consistently act as fear inhibiting or ‘Fear-Off' neurons during behaviour. We use cell-type-specific optogenetics and chemogenetics (DREADDs) to modulate activity in this population during behaviour to block or enhance fear extinction. Dissociated Thy1-eYFP neurons are isolated using FACS. RNA sequencing identifies genes strongly upregulated in RNA of this population, including *Ntsr2, Dkk3, Rspo2* and *Wnt7a*. Pharmacological manipulation of neurotensin receptor 2 confirms behavioural effects observed in optogenetic and chemogenetic experiments. These experiments identify and validate *Ntsr2*-expressing neurons within the BLA, as a putative ‘Fear-Off' population.

## Abstract (web summary)

Activation of Thy-1 expressing neurons in the basolateral amygdala (BLA) has been shown to result in inhibition of fear. Here the authors present a comprehensive workflow to identify genes that are upregulated in this population and can be pharmacologically targeted to enable fear extinction.

## Graphical abstract

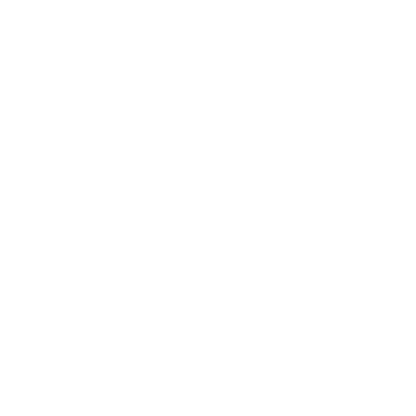

Overexpression of learned fear underlies many neuropsychiatric disorders such as phobia, panic disorder and post-traumatic stress disorder. Hallmark symptoms of post-traumatic stress disorder such as re-experiencing memories of traumatic events, intrusive thoughts and hyper-arousal are likely precipitated by the over-consolidation of and failure to extinguish learned fear associations^1^,^2^. Classical fear conditioning allows for the controlled study of neuronal processes mediating associative fear. Pavlovian fear conditioning is a learning paradigm wherein an initially neutral conditioned stimulus (CS; for example, a tone) is paired with an aversive unconditioned stimulus (US; for example, a mild footshock). After several CS+US pairings, mice display threat responses (also referred to as fear behaviours) upon presentation of the tone CS alone, here, fear behaviours are measured by quantifying freezing (when the mouse ceases all non-homeostatic motion)^3^. During fear extinction, the CS tone is presented in the absence of any US shock until the threat responses return to baseline^3^,^4^,^5^,^6^. The amygdala is well known to have an essential role in most fear behaviours, specifically in the acquisition and extinction of learned associations^7^,^8^. Here we will refer to fear conditioning as the training phase where CS/US associations are acquired, fear extinction as the early period where CS presentations in the absence of US lead to decrement in freezing, and fear extinction retention as a later period where additional CS presentations measure the strength of retention of initial extinction event and act as additional extinction events. It should be noted that fear expression, fear extinction and extinction retention are overlapping processes, where we are measuring the balance of signalling processes rather than unitary elements acting independently, thus early fear extinction measures primarily fear expression to CS and extinction, while late fear extinction measures primarily extinction expression and consolidated retention of previous extinction learning.

Essential to understanding the opposing processes of fear acquisition and its extinction is the identification of neuronal circuits mediating each process^9^,^10^,^11^,^12^,^13^. Furthermore, development of rationally designed pharmaceuticals that act directly on fear-inhibiting circuitry depends on discovering the molecular identities of neuronal populations that specifically mediate fear extinction. Within the basolateral amygdala (BLA), two electrophysiologically distinct populations of principal neurons neurons have been described, one increasing firing in response to the CS to signal fear conditioning and expression (Fear-On) and the other increasing its firing to the CS during fear extinction (Fear-Off); differences in the activity in these populations have been shown to accurately predict freezing behaviour of mice^10^.

Identifying the molecular identities of these populations represents a major step towards full characterization of this circuit^14^. Importantly, we have previously electrophysiologically and functionally described a population marked by the Thy1 expression cassette that contains elements of the Fear-Off population^15^. Thy1-channelrhodopsin-2 (Thy1-ChR2-eYFP) and Thy1-YFP lines mark a population of glutamatergic pyramidal neurons within the BLA, with significant specificity for the anterior basal amygdala^16^. Optogenetic activation of this population leads to the inhibition of a population of central amygdala (CeA) neurons and shunts excitatory drive from the lateral amygdala (LA). Behaviourally, brief activation of these neurons co-terminating with an unreinforced CS during extinction learning leads to enhanced extinction retention.

It is critical to further describe the behavioural and molecular phenotypes of this important population of cells, and in doing so identify a cell-type-specific pharmacological substrate for enhancing fear inhibition. This work presents a work-flow to achieve this goal that may be applied to any genetically identified neuron population. Additional Thy1 transgenic lines are further functionally dissected with optogenetics and chemogenetics, demonstrating the necessity of these neurons for fear extinction learning. Furthermore, we characterize the RNA and protein expression patterns of this population, and identify neurotensin receptor 2 (NTSR2) as one of several possible pharmacologically targetable markers of this fear-inhibiting neural circuitry. Our data suggest that pharmacological modulation of NTSR2 activity during behaviour recapitulates effects observed from manipulation of Thy1 neurons, suggesting a consistent role for this neural population in fear inhibition.

## Results

### Thy1 marks consistent population of BLA neurons

**Figure 1:**
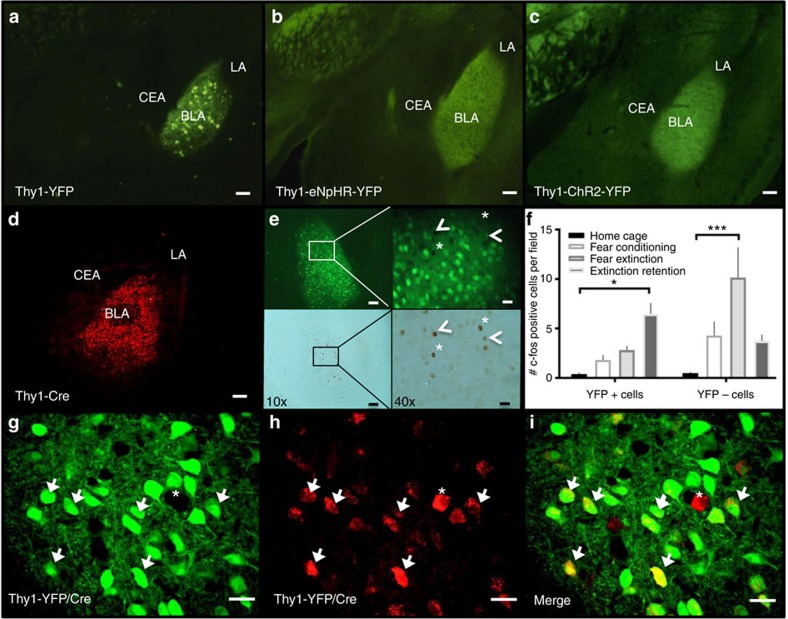
Thy1 lines mark BLA population that is active during expression of fear extinction. (**a**) Thy1-eYFP, (**b**) Thy1-eNpHR, (**c**) Thy1-ChR2, each have strong expression enriched in the BLA. (**d**) Cre recombinase driven by the Thy1.2 cassette in the Thy1-cre mouse, when visualized via infusion of AAV- hSyn-DIO-rM3D(Gs)-mCherry, marks the same regional population as marked by the Thy1-eYFP mouse. (**e**) Representative images of a field of view analysed for c-fos staining in the BLA of Thy1-YFP mouse after fear expression. Co-localization was manually scored at × 40 magnification, × 10 images are for reference. Upper panels: YFP+ expression in BLA of examined Thy-1 YFP mice. Green neurons in upper panels are YFP+ neurons while black c-fos+ nuclei are depicted in lower panels. Fields for analysis were chosen at random within strongly YFP expressing area. Fields of were visually inspected for co-labelling of c-fos and YFP. Example neurons identified: arrowhead indicates c-fos+/YFP+ cell, while asterisk indicates c-fos+/YFP− cell. (**f**) Quantification of co-labelling of YFP or non-YFP-marked cells with c-fos in Thy1-eYFP mouse. Counts represented as number of positive events per field analysed. BLA sections were analysed after (1) untrained home cage controls (HC), (2) fear conditioning (FC), (3) fear extinction (FE) or (4) fear extinction retention (FR). Thy1-eYFP+ neurons are c-fos+ significantly more often after fear extinction testing versus home cage, while eYFP-marked neurons stain for c-fos significantly more often during fear expression (two-way ANOVA, F_(3,40)_ *P*=0.0017, when significant main or interaction effects were found by the ANOVAs, Sidak's *post hoc* tests were carried out to locate simple effects. Sidak's multiple comparisons versus home cage: *P<0.05, *P<0.005, error bars indicate ±s.e.m. Sidak's multiple comparison *post hoc* analysis: YFP+ HC versus YFP− FE *P*<0.05, YFP+ FC versus YFP− FE *P*<0.05, YFP+ FE versus YFP− FE: *P*<0.05, YFP− HC versus YFP− FE *P*<0.05, YFP− FE versus YFP− FR: *P*<0.05, error bars indicate mean±s.e.m.). (**g**–**i**) Infusion of AAV-EF1a-DIO mCherry into double transgenic Thy1-eYFP/Thy1-Cre mouse allows co-localization of neurons marked by each transgene. Arrows indicate neurons with co-localization. Asterisks indicate neuron marked only by mCherry. Scale bar, 100 μm (**a**–**d**, **e** upper left and lower left); scale bar, 20 μm for (**g**–**i**, **e** upper right and lower right).

Expression patterns were assessed across: Thy1-eYFP line-H (Fig. 1a)^16^,^17^, Thy1-eNpHR line 2 (Fig. 1b)^18^,^19^, Thy1-ChR2-EYFP line 18 (Fig. 1c)^20^ and Thy1-Cre line 1 (Fig. 1d)^21^. All lines produce high levels of transgene specifically within the BLA primarily the anterior BLA, sparing the LA, CeA and more caudally the basomedial amygdala. The Thy1-eYFP line was crossed with the Thy1-Cre line and infused unilaterally into the BLA with a Cre-dependent reporter virus. The resulting YFP fluorescence from the Thy1-eYFP line and mCherry from Thy1-Cre line exhibited strong overlap. Of mCherry (Thy1-Cre) labelled cells in the BLA, 93% were also YFP labelled demonstrating strong coherence in the identity of this population marked across Thy1 lines (Fig. 1g–i). Because the rate of infected neurons is likely <100%, it is not possible to positively verify the proportion of YFP+ neurons that concurrently express Cre in this system; however, these data suggest that the Thy1-Cre+ population is contained almost entirely within the Thy1-YFP population. The reporter virus used here is extremely sensitive to recombination and may be recombined by a single molecule of Cre thereby detecting expression levels of Cre that are subthreshold for significant transgene expression in other lines (discussed in greater depth in supplement). For similar reasons, when the Thy1-Cre mouse line was crossed with reporter lines, as was done in initial characterization, a much larger population of cortical neurons is marked. Alternatively, this may suggest promiscuous expression during development with a more constrained pattern at adulthood; each possibility is discussed at greater length in Supplementary Discussion^21^. Co-localization between Thy1-eNpHR and other lines was not possible with this scheme because the transgene product is membrane bound making visualization of individual cell bodies within the densely populated BLA very difficult.

### Separate neuron populations active during fear processes

Thy1-ChR2-eYFP-expressing neurons of the BLA appear to contain a ‘Fear-Off' controlling population (Jasnow *et al*.^15^). To verify that the Thy1-eYFP-marked population plays the same role in behaviour, each of four cohorts of Thy1-eYFP mice (home cage, fear conditioned, fear extinction and extinction retention) was put through the classical auditory fear-conditioning paradigm and sacrificed immediately following selected session^15^. All tissue was processed and stained for c-Fos expression using immunohistochemistry (Fig. 1e). Following fear expression/extinction training, a significant increase in c-Fos was observed in the YFP− but not the YFP+ population. However, following the extinction retention test, a significant increase in c-Fos was observed in the YFP+ but not the YFP− population, suggesting that the BLA is involved in both fear expression and extinction, but the Thy1 population is selectively recruited during extinction and extinction retention. These data agree with previous the data, suggesting that Thy1+ neurons may be crucial for the encoding of fear extinction; from this lens, we would expect the Fear-On (Thy1-) population to be primarily active during the early phases of fear extinction, while the Fear-Off (Thy1+) population should increase activity as extinction progresses, thus during extinction retention session (which is also an additional extinction session) the YFP+ population is primarily active, while the YFP− population is less activated. These behaviours do overlap during extinction and extinction retention, thus a long extinction test was used, so that during the extinction retention session the mouse is well within the extinction expression phase and a preponderance of the c-fos staining is observed in the Thy1+ population during this session.

### Electrophysiological characterization of Thy1-eNpHR neurons

**Figure 2:**
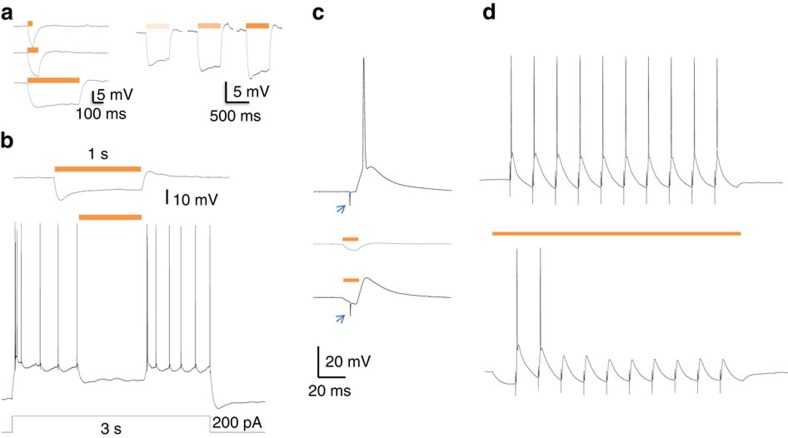
Halorhodopsin inhibition of BLA Thy-1 neurons. (**a**) Light activation of halorhodopsin (593 nm) induced membrane hyperpolarization in a Thy-1 neuron; strength of hyperpolarization is time and intensity-dependent. (**b**) Halorhodopsin activation abolished inward current injection-induced firing; (**c**) upper, electrical stimulation of lateral amygdala induced action potential in a BLA Thy-1 neuron; middle, light evoked membrane hyperpolarization in Thy-1 neuron; bottom, the action potential was abolished by light illumination; arrowheads indicate stimulus artifact from LA stimulation. (**d**) Upper, a train of action potentials induced by 10 Hz electrical stimulation of LA, lower, the firing was reduced by concurrent halorhodopsin activation. Delays in action potential suppression are likely due to transient and complex dynamics of intrinsic ion channels during eNpHR facilitated hyperpolarization. Similar delays were found in original characterizations of Thy1-NpHR mouse lines. For additional characterization of NpHR and eNpHR in Thy1 mouse lines, please see refs 18, 19.

To confirm that eNpHR is capable of inhibiting Thy1 neurons, we measured the electrophysiological properties and light response of Thy1-eNpHR-eYFP neurons in BLA slice. As expected, we found strong laser illumination (wave length 593 nm, 3.6–10 mW mm^−2^) induced hyperpolarization of BLA principal neurons(Fig. 2a). Laser illumination was sufficient to inhibit current injection-induced spiking in eNpHR-YFP-expressing neurons (Fig. 2b). To confirm that transgene expression has no adverse effects on normal neuron firing in response to extrinsic inputs, normal action potential generation in eNpHR-expressing neurons was measured with electrical stimulation of LA (Fig. 2c upper). The action potential was effectively abolished with laser illumination (Fig. 2c, lower). Trains of action potentials were reliably produced by 10 Hz electrical stimulation (Fig. 2d, upper). These action potentials were effectively blocked by laser illumination (Fig. 2d, lower). Taken together these results demonstrate that in the absence of laser light, eNpHR-eYFP-expressing neurons have normal responses to synaptic input and laser light is sufficient to consistently hyperpolarize these neurons blocking the majority of generated action potentials.

### Optogenetic silencing of Thy1 neurons

**Figure 3:**
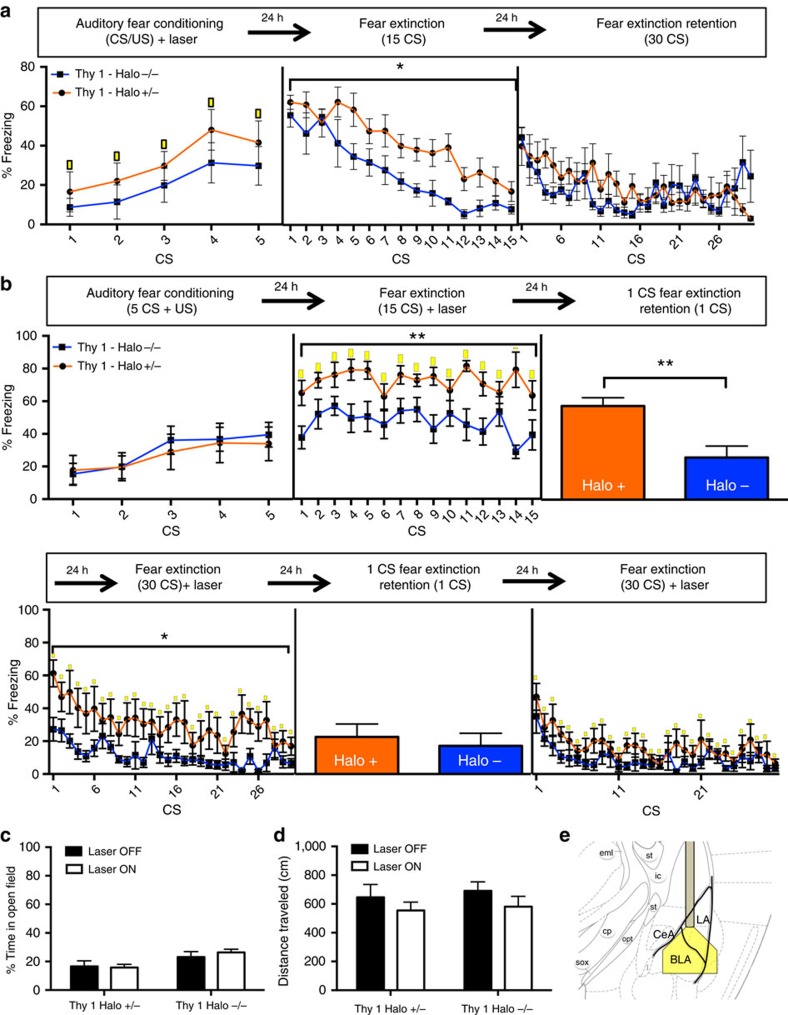
*In vivo* inhibition of Thy1 neurons. Inhibition of Thy1 neurons enhances fear consolidation and fear expression, while blunting fear extinction consolidation. BLA neurons of mice expressing halorhodopsin under the control of the Thy1.2 expression cassette were silenced during select phases of fear conditioning to determine their precise role in behaviour. This change in fear behaviour was not caused my changes in anxiety-like behaviour. (**a**) Silencing BLA Thy1 neurons during weak fear acquisition results in no significant within-session changes in behavior; however, 24-h later mice carrying the Thy1-halorhodopsin gene express significantly more fear during fear extinction (two-way Repeated measures (RM) ANOVA, F_(1,14)_=5.827 ,*P<0.05). (**b**) Mice weakly fear conditioned in the absence of laser stimulation show no within-session behavioral differences; however, 24-h later when laser stimulation is applied during CS-on period of extinction, mice carrying the Thy1-halorhodopsin gene express significantly more fear (two-way RM ANOVA, F_(1,12)_=10.08,**P<0.01). Thy1-halorhodopsin carrying mice exhibit significantly enhanced fear 24-h later in the absence of laser stimulation (Student's *t*-test, **P<0.01). 24-h later, enhanced freezing is measured throughout the extinction retention session when laser stimulation is provided during CS-on period (two-way RM ANOVA, F_(1,11)_=7.75, *P<0.05). 24-h later no difference in freezing to CS is detected in the absence of laser stimulation. A final extinction session with laser stimulation during CS presentation reveals no differences in freezing. (**c**) Inhibition of BLA Thy 1 neurons was not accompanied by any change in anxiety-like behavior or (**d**) ambulation. Thy1-halorhodopsin carriers spent the same amount of time in the centre of an open field as controls whether laser light was on or off. Thy1-halorhodopsin carriers travelled the same distance whether laser light was on or off. (**e**) Schematic of optical fibre placed dorsal to the BLA providing yellow laser illumination during experimental procedures. Light is estimated to maintain >10 mW power within 0.5 mm of the fibre optic tip. In all panels error bars indicate mean±s.e.m.

Mice were implanted with optical fibres housed within ceramic ferrules unilaterally, just dorsal to the amygdala, aimed at the BLA of Thy1-eNpHR carrying and non-carrier controls. Fibre tip placement was confirmed using Cresyl violet staining and any mouse whose fibre was not placed over BLA was excluded from analysis (Supplementary Fig. 1a). Mice were mildly fear conditioned to avoid ceiling effects (5CS+US, 0.4 mA US foot shock). Yellow (593 nm) light was tonically applied throughout the 30-s CS (Fig. 3a). Inhibition of Thy1-eNpHR neurons during acquisition caused no changes in within-session behaviour; however, during 15 CS fear extinction session (considered a partial/sub-optimal extinction session) in the absence of yellow laser stimulation, Thy1-eNpHR carriers expressed significantly more freezing throughout the session. These results suggest that during fear acquisition, the BLA Thy1 population acts as a brake on fear learning and when it is silenced, fear acquisition is enhanced. Importantly, Thy1-eNpHR carriers and non-carriers that are cannulated and trained in an identical manner, but never receive any laser light, exhibit identical levels of freezing, confirming that any behavioural differences are caused by light-mediated silencing (Supplementary Fig. 2).

To determine whether BLA Thy1 neurons play a role during fear expression, a naive cohort of Thy1-eNpHR-expressing and non-carrier littermates received optic fibre implants, and was fear conditioned without any laser application (Fig. 3b). During short 15 CS fear extinction, laser light was applied throughout the CS. Thy1-eNpHR carriers expressed significantly more fear throughout the session starting from the first tone, suggesting that inhibition of BLA Thy1 neurons reversed inhibition of the fear trace allowing for enhanced expression. Thy1-eNpHR carriers displayed significantly more freezing during 1 CS unstimulated expression test, suggesting that previous inhibition had not only enhanced fear expression, but blunted extinction as well. Mice were further extinguished (retention) to 30 CS's with laser application during the CS. Thy1-eNpHR carriers expressed enhanced fear throughout this fear extinction session. By the end of the session both groups of mice extinguished to baseline freezing levels. Finally, once a fearful association is extinguished, the inhibition of BLA Thy1 neurons during the final extinction session is not sufficient for the reinstatement and does not drive spontaneous fear expression.

These changes in fear behaviour were not accompanied with any anxiety or locomotor effects indicated by similar time spent in the centre of an open field (Fig. 3c) and distance travelled (Fig. 3d) by both groups, regardless of whether laser light was on or off. Placement of fibre tip was confirmed by Cresyl violet staining (Supplementary Fig. 1b).

### Chemogenetic activation of Thy1 neurons

**Figure 4:**
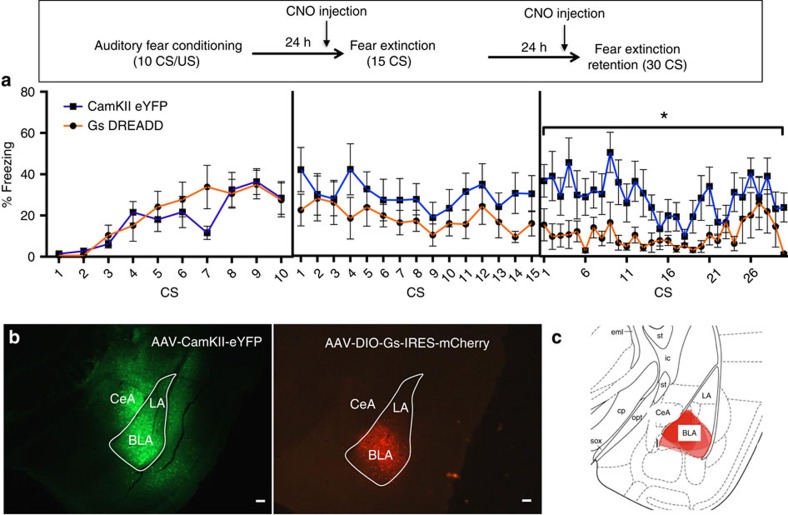
Enhancing excitability of BLA Thy1 neurons using DREADDs. Mice harbouring the Thy1-cre gene were infected with AAV-EF1a-DIO-rM3D(Gs)-mCherry or control virus AAV-CamKII-eYFP. Mice were auditory fear conditioned then administered clozapine-*N*-oxide 30 min before fear extinction and fear extinction retention sessions. (**a**) Tonic enhancement of excitability of BLA Thy-1 neurons during fear extinction enhances consolidation of learned extinction as measured 24-h later during fear extinction session(two-way RM ANOVA, F_(1,14)_=6.200 , *P<0.05). (**b**) Location of Gs DREADD or control virus is visualized using mCherry or YFP tag. Cre-dependent recombination causes expression of Gs-DREADD-mCherry to be isolated to BLA Thy1 neurons, while control virus has widespread expression throughout amygdala. Scale bar, 100 μm. (**c**) Depiction of aggregated expression patterns in left BLA of strong cre-dependent mCherry expression at −1.8 DV in AAV-EF1a-DIO-rM3D(Gs)-mCherry infused animals. Scale bar, 100 μm. In all panels error bars indicate mean±s.e.m.

The Thy1-Cre line was utilized to modulate activity in Thy1 neurons using chemogenetics (Fig. 4a). Designer receptors exclusively activated by designer drugs (DREADDs) are modified G-protein-coupled receptors that are activated exclusively by an otherwise inert ligand, clozapine-*N*-oxide (CNO)^22^,^23^,^24^. Here, we use a Gs-coupled DREADD that is known to increase intracellular cAMP levels and enhance excitability when CNO is bound^25^,^26^. Thy1-Cre mice were infected bilaterally with either AAV-DIO-Gs-DREADD-IRES-mCherry or AAV-CaMKII-eYFP. Expression of Gs DREADD infection is isolated to BLA; however, the pan-excitatory neuron expressing CaMKII-eYFP virus resulted in strong reporter expression throughout the amygdala (Fig. 4b). Mice were fear conditioned (0.65 mA CS, 10CS+US pairings). The Friend virus type-B (FVB) background of the Thy1-Cre mice is not a high-freezing line, thus a stronger fear-conditioning paradigm (10CS+US) was necessary to achieve sufficient levels of freezing for experimental manipulation. Mice were injected with CNO twice at 1 mg kg^−1^, once 30 min before fear extinction and again 24 h later before the extinction retention session^26^. Mice expressing the Gs-DREADD expressed similar levels of fear to the tone during the initial 15 CS fear extinction test; however, 24 h later during a 30 CS extinction retention session they expressed significantly less fear (Fig. 4a).

These results suggest that tonic enhancement of excitability in BLA Thy1 neurons during the extinction session is not sufficient for within-session changes in behaviour. However, enhanced excitability is sufficient to enhance the fear extinction consolidation, resulting in overall marked decreases in fear expression during the subsequent fear extinction retention session. However, it is possible that CNO administered before extinction retention enhances extinction expression, despite having no within-session effect during extinction. Taken together these results further support the hypothesis that the BLA Thy1 neurons are a ‘Fear-Off' population.

### Isolation and molecular characterization of Thy1 neurons

**Figure 5:**
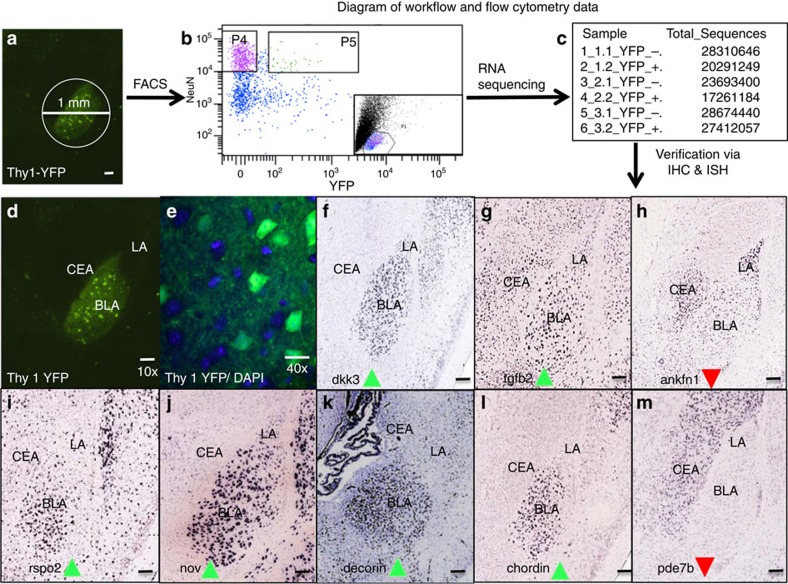
Workflow describing FACS sorting and sequencing of RNA of Thy1-eYFP cell bodies, cell-type-specific RNA sequencing and identification of differentially regulated gene transcripts. (**a**) Schematic indicating location of tissue punch. The bilateral amygdala of Thy1-eYFP mice were obtained via bilateral 1 mm punches centred over the basolateral amygdala. These tissue samples were dissociated into single-cell suspensions, fixed and stained for neuronal marker, NeuN. Cell suspensions were sorted based on fluorescent profiles and NeuN+/YFP− and NeuN+/YFP+ populations were collected for RNA analysis. (**b**) Representative scatterplot generated during FACS. Box P4 indicates NeuN+/YFP− events, Box P5 indicates NeuN+/YFP+ events. Inset: representative FSC/SSC; neuron cell bodies were present in small population at bottom. (**c**) Chart demonstrating total number of sequences yielded with RNA-seq collected by each sample with FACS. (**d**) Image of YFP expression in Thy1-YFP mouse amygdala Scale bar, 100 μm. (**e**) × 40 magnification of Thy1-YFP expression demonstrating that only a subset of cell bodies (indicated by DAPI; blue) express YFP (Green) scale bar, 20 μm. (**f–m**) Examples of genes identified as differentially regulated. RNA sequencing yielded a list of hundreds of differentially regulated genes. After refinement using a predetermined set of exclusion criteria (Supplementary Fig. 3) RNA expression patterns of genes were examined using the Allen Brain Map for enriched expression within the BLA. Green arrows indicate genes up regulated in Thy1-eYFP neurons versus all other neurons (*Dkk3, Tgfb, Rspo2, Nov, Dcn* and *Chrd*). Red arrows indicate genes down regulated in Thy1-eYFP neurons versus all other neurons (*Ankfn1* and *Pde7)*. Image Credit: Allen Institute^31^,^32^,^33^,^34^,^35^,^36^,^37^. Scale bar, ∼100 μm.

We next wished to further understand the molecular components that differentiate the Thy1+ cells from the Thy1− cells, hypothesizing that these cells may underlie genetic differences in the Fear-On versus Fear-Off BLA populations. Furthermore, to rationally identify pharmaceuticals that selectively modulate known fear-controlling circuits, it is advantageous to know the molecular expression profiles of targeted neurons. To approach this problem, we isolated Thy1 neurons using fluorescence-activated cell sorting (FACS), then interrogated their molecular identity using RNA sequencing. To obtain purified samples of Thy1-eYFP neurons, 1 mm punches of tissue centred over BLA were taken from Thy1-eYFP-expressing mice and pooled (Fig. 5a). Tissue was dissociated into a single-cell suspension and fixed for staining with NeuN antibody and Hoechst; this allowed identification and isolation of specific neuronal cell bodies^27^,^28^,^29^. Voltages and gates were set using wild-type C57/B6 stained and unstained controls. Samples were analysed for forward scatter and side scatter characteristics. A large portion of NeuN positive, Hoechst positive events were found in a small population near the bottom of the plot, so this population, corresponding to cell bodies, was chosen for further interrogation (Fig. 5b, inset). We collected two populations, one strongly NeuN positive, strongly YFP negative and the other strongly NeuN positive, strongly YFP positive (Fig. 5b). Although this method reduced the total yield, it increased the purity of collected neurons. Overall, of all cell bodies interrogated, 40% were NeuN positive, while only ∼2% were NeuN and YFP double positive. Collected cells were confirmed to be either single or double positive using microscopy. Following isolation, RNA was immediately collected from samples and later sequenced.

RNA sequencing revealed thousands of differentially regulated genes between Thy1-eYFP and other neurons. This list was then curated based upon a set of criteria demanding the gene must be highly significant controlling for false discovery rate (*q*<0.05), and differentially regulated at a fold change >2^0.5^ (Supplementary Fig. 3). Furthermore, a list of gene hits meeting these inclusion criteria was input into the drug-able genome database (DGIdb) and interaction partners were searched for from expert curated lists, thus allowing for identification of gene targets that have high-quality drug interaction partners. These top curated genes were then examined for expression patterns within the BLA, using the Allen Brain Atlas or the literature.

Genes found to have increased expression in Thy1 neurons and the BLA include: *Dkk3, Tgfb, Rspo2, Nov, Dcn* and *Chrd*; genes found to have relatively decreased expression in Thy1 neurons and the BLA include *Ankfn1* and *Pde7b* (Fig. 5f–m). Data are shown only from differentially regulated genes that had high-quality coronal images in the Allen Brain Atlas^30^,^31^,^32^,^33^,^34^,^35^,^36^,^37^. Other genes that did not have high-quality coronal *in situ* hybridization images available through the Allen Institute were examined in the literature for messenger RNA (mRNA) and protein expression. Several genes, notably *Ntsr2*, were determined to have enriched or depleted expression in BLA and thus met inclusion criteria^38^,^39^. RNA sequencing expression data were validated using quantitative PCR (qPCR) for select genes of interest. Follow-up qPCR supported a strong upregulation of *YFP*, *Thy1* and *Ntsr2* mRNA in YFP+ sample (Supplementary Fig. 4). Results from qPCR validation performed on RNA used for sequencing replicate data demonstrated that the fluorescent Thy1 neurons were successfully sorted, and that RNA sequencing indeed identified differentially expressed genes that are selectively upregulated in these neurons.

This methodology strongly biases our results towards genes that are selectively upregulated in Thy1 neurons to the exclusion of genes with equal population-specificity, but less regional specificity. Our goal is to discover putative drug targets selectively acting on the ‘Fear-Off' population within the BLA. This goal is most efficiently achieved by narrow consideration of genes that are highly upregulated in putative Fear-Off populations.

**Figure 6:**
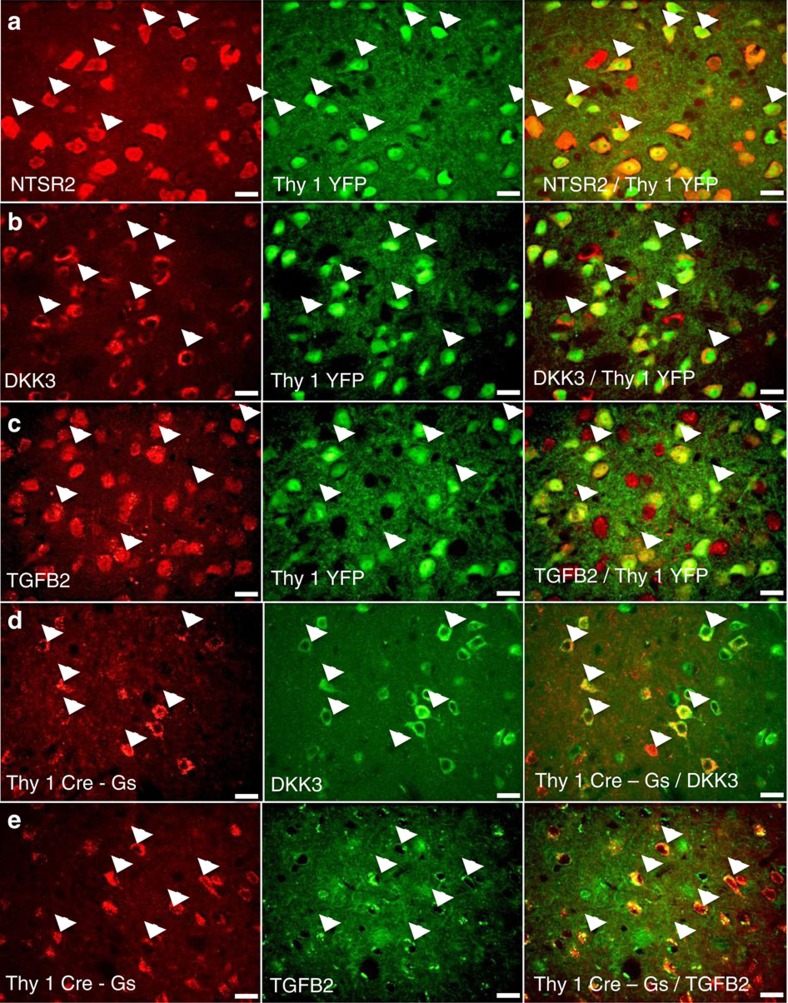
Molecular characterization of basolateral amygdala Thy1 neurons. Examination of co-localization with protein products of identified differentially expressed genes. Molecular characterization of Thy1-eYFP-expressing neurons of the basolateral amygdala was completed on tissue from Thy1-eYFP line H mice. Coronal sections were stained for protein of interested using immunohistochemistry visualized using secondary antibodies emitting in the red spectrum. Thy1-eYFP strongly co-localizes with (**a**) NTSR2, (**b**) DKK3 and (**c**) TGFB2. Tissue taken from Thy1-Cre-expressing animals infused bilaterally with AAV-EF1a-DIO-rM3D(Gs)-mCherry allows visualization (red) of Thy1-Cre-expressing neurons. Coronal sections of this tissue was stained above for (**d**) DKK3 and (**e**) TGFB2, revealing that these markers co-localize to similar extent with both Thy1-eYFP and Thy1-Cre. Scale bars, 20 μm.

Protein expression patterns of six genes selectively upregulated in Thy1-eYFP neurons were next examined using immunohistochemistry in tissue from Thy1-eYFP mice (Fig. 6; Supplementary Fig. 5). Using confocal microscopy, the degree of co-localization between the protein of interest and Thy1-eYFP neurons was examined quantitatively. Images were taken in regions of high Thy1-eYFP density with equal numbers of volumes analysed anteriorly and posteriorly. Genes examined are: *Ntsr2, Dkk3, TgfB2, Rspo2, Wnt 7a* and *Dcn.* Across all markers examined, none had >1% of YFP-positive cells that did not co-localize with the marker of interest (Supplementary Fig. 6). All genes exhibited staining in some cells that were not YFP positive, suggesting that the Thy1-eYFP population may be marking a majority sub-population of a larger population with a common protein expression profile.

To confirm that these apply across Thy1 transgenic lines, tissue previously used for the above chemogenetic experiments was stained using antibodies against Dkk3 and TGFB2, and co-localization was measured with Gs-DREADD expression. A majority population of co-labelled cells was revealed with a minority population of cells single labelled for the gene of interest and ∼1% Thy1-Cre-marked neurons remaining unlabelled. Although all markers had high levels of co-localization in the regions analysed, *Ntsr2* and Dkk3 displayed regional homology across all amygdala sections examined (Supplementary Fig. 7). Furthermore, the availability of high-quality agonist and antagonist drugs, acting specifically at NTSR2 prompted the further investigation of NTSR2 and its role in fear learning^40^,^41^.

### Pharmacological manipulation of NTSR2

**Figure 7:**
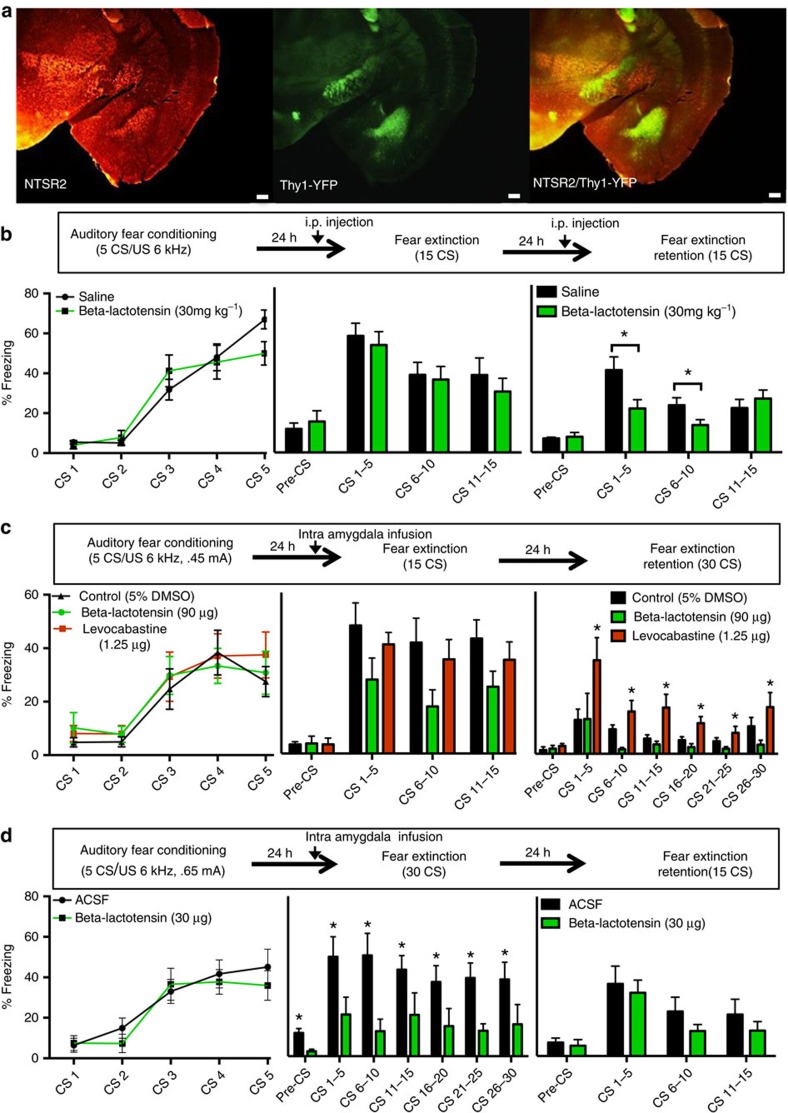
Modulating neurotensin receptor 2 activity alters fear expression and consolidation. (**a**) Regionally NTSR2 has an overlapping regional expression pattern to Thy1-eYFP along the rostral-caudal axis of the amygdala. Scale bar, 200 μm. (**b**) Beta-lactotensin, a NTSR2 agonist, was given i.p. at 2 h before fear extinction and fear extinction retention sessions. This enhanced consolidation of fear extinction as measured by significantly decreased fear expression during first 10 CS presentations of fear extinction retention session (two-way RM ANOVA, F_(1,19)_=5.39, *P<0.05). (**c**) Intra-amygdala infusion of 90 μg per hemisphere beta-lactotensin 30 min before fear extinction test yields near significant decreases in within-session freezing expression (two-way RM ANOVA, F_(2,32)_=2.707, *P*=0.085); however, infusion of 1.5 μg per hemisphere levocabastine causes no within-session effects, but blunts fear extinction consolidation as measured by increased freezing during fear extinction retention session (two-way RM ANOVA, F_(2,20)_=6.633, *P*<0.01, Dunnetts multiple comparison test versus control *P<0.05). (**d**) To ensure prior training did not cause previous results, and to test a lower dose of drug, naive animals received bilateral infusion of 30 μg per hemisphere beta-lactotensin or saline control. Infusion of this lower dose of beta-lactotensin again resulted in dramatic reductions in within-session freezing (two-way RM ANOVA, F_(1,9)_=13.28, *P<0.01). In all panels error bars indicate mean±s.e.m.

The above evidence suggested that the NTSR2 is highly expressed within the Thy1 neurons within the BLA, sharing consistent regional and cellular specificity (Fig. 7a). To assess NTSR2 as a target for actuating pharmacological manipulation of fear behaviours, a selective NTSR2 agonist, beta-lactotensin, was acquired and its effects on fear expression measured. Mice were fear conditioned (Fig. 7b) and 24-h later, 2 h before fear extinction, mice were injected intraperitoneally (i.p.) with either saline or beta-lactotensin (30 mg kg^−1^)^42^. No within-session effect of beta-lactotensin on fear expression was detected; however, during extinction retention, 2 h after a second injection, a significant decrease in freezing was detected during the first ten tone presentations in mice receiving beta-lactotensin administration. These data suggest that beta-lactotensin may act to enhance NTSR2 activity, stimulating the amygdala ‘Fear-Off' population and enhancing fear extinction consolidation and expression of extinction.

We next wished to confirm that the behavioural effects detected following peripheral administration of beta-lactotensin reflect changes in activity of BLA NTSR2-expressing neurons. Thus, we performed another experiment directly comparing agonists and antagonists at this receptor with targeted intra-amygdala injections. Beta-lactotensin and levocabastine, a selective NTSR2 antagonist, were applied directly to the amygdala in separate cohorts of mice before fear extinction (Fig. 7c). Mice were cannulated bilaterally with cannulae aimed so that the injector tip rested just above the BLA. On the basis of our halorhodopsin experiments, a weak fear conditioning (0.4 mA US) was necessary to avoid ceiling effects. Twenty-four hours later mice were infused bilaterally into the BLA with beta-lactotensin (90 μg per hemisphere), levocabastine (1.5 μg per hemisphere) or vehicle (5% dimethylsufoxide (DMSO) in saline, needed to maintain common vehicle), 30 min before fear expression. Mice infused with beta-lactotensin had a strong, but non-significant trend towards reduced freezing throughout the fear extinction session, suggesting that activating NTSR2 in the BLA is sufficient to decrease fear. There was no within-session effect of levocabastine infusion during fear expression; however, during the fear extinction retention session, mice that previously had been infused with levocabastine froze significantly more than control. To confirm that observed effects on freezing were not due to changes in anxiety-like behaviour or locomotion, mice were infused with their original drug and placed in an open-field chamber for 10 min. No differences between groups were detected in either time spent in centre or distance travelled (Supplementary Fig. 8). Together, these data suggest that inhibiting NTSR2 within the BLA is sufficient to block fear extinction resulting in more fear expression.

Although beta-lactotensin has high affinity for NTSR2, it has 10-fold lower affinity for NTSR1 as well. To confirm that observed behavioural changes were not due to off-target agonism of the NTSR1 mice a separate, naive cohort of mice were cannulated and fear conditioned at a higher shock intensity (0.65 mA; Fig. 7d)^43^. Mice were infused bilaterally with beta-lactotensin at a threefold lower concentration (30 μg per hemisphere) or sterile saline 30 min before fear extinction session. Mice that received infusion of beta-lactotensin expressed significantly less fear than their saline-infused counterparts. Interestingly, during the pre-CS period the group infused with beta-lactotensin showed less fear compared with controls as well, suggesting that the agonism of the NTSR2 causes generalized inhibition of fear expression (unpaired *t*-test, *P*<0.01). Twenty-four hours later mice were subjected to another fear extinction retention session without drug, and no detectable differences were measured between groups, possibly due to the previous full fear extinction test resulting in a floor effect. Overall, these experiments show that activation of NTSR2 within the amygdala is sufficient to decrease fear expression.

### Examination of projection patterns of BLA Thy1-Cre neurons

To understand how the Thy1/NTSR2 population fits into a larger fear-controlling circuitry, it is necessary to uncover the specific projections of this population. Thy1-Cre-expressing mice were infused unilaterally with cre-dependent (AAV-DIO-GFP) virus into the BLA at different anterior to posterior positions (−1.0, −1.5, −2.0). After 3 months, animals were killed and the patterns of fluorescent protein expression were examined descriptively throughout forebrain structures for regions of apparent strong and weak YFP fluorescence. Labelled cell bodies were found primarily within 0.5 mm along the A/P axis of the site of infusion (Supplementary Figs 9f, 10e,f and 11c,d). Examination of patterns made by filled terminals were made with no *a priori* hypotheses and revealed strong projections from BLA Thy1 neurons to several regions, including nucleus accumbens (NAc) core and shell, and bed nucleus of the stria terminalis (BNST; Supplementary Figs 9b,c, 10a,c,g and 11b). Both of these regions mediate, among many other functions, reward and positive valence. Additional projections were found to the prefrontal cortex (with some specificity for the Infralimbic cortex (IL)), anterior insula, contralateral BLA and contralateral medial intercalated nuclei (mITC)(Supplementary Figs 9a,d, 10b,d and 11a). These regions, especially IL and mITC have been strongly implicated, as mediating extinction and fear suppression. Importantly, although anterior infusions spare posterior cell bodies and posterior infusions spare anterior cell bodies, few discrepancies were found in the patterns of projections between infusion sites. Importantly, and perhaps consistent with the apparent role of the Thy1-marked cell in Fear-Off regulation, there is little apparent projection to CeA from these labelled cells (Supplementary Figs 9d,e, 10d–f and 11c).

When compared with cre-dependent tracing, biotinylated dextran amine infused anteriorly into the BLA (−1.5 A/P) results in similar projection patterns; notably, projections are very weak to the CeA as observed in Thy1-eYFP and Thy1-Cre animals (Supplementary Fig. 12, CeA labelling in 12A is due to over-flow from infusion). Projections to IL and BNST are observed as well. However when biotinylated dextran amine is infused posteriorly (−2.5 AP) there are strong projections observed to all parts of the CeA, as well as BNST and anterior insula (Supplementary Fig. 13). For additional discussion of Thy1-Cre expression, see Supplementary Discussion (Supplementary Figs 14 and 15). Taken together, these data suggest that Thy1 ‘Fear-Off' neurons conform to projection patterns of the majority of rostral BLA neurons; however, they maintain their segregated projections more caudally, where CeA projecting neurons that likely have an alternative role in behaviour can be found as well.

## Discussion

Data presented here: (1) further identify and characterize a functional role for the Thy1-marked amygdala neural population in behaviour, (2) characterize the molecular profile of this population, (3) characterizes its circuit connectivity and (4) uses that information to identify compounds that directly modulate activity of the population *in vivo*. This represents an executable methodology for behavioural and molecular characterization of a neuron population leading to identification of pharmacological agents that act *in vivo.*

Four Thy1 transgenic lines mark consistent regional and cellular populations specifically within the BLA. Populations marked by these lines maintain consistent roles in learned fear behaviour as indicated by their recruitment during fear extinction (Thy1-eYFP), sufficiency of tonic enhancement of excitability for augmented fear extinction consolidation (Thy1-Cre), and necessity of activity for fear inhibition and extinction (Thy1-eNpHR). Previously, we have demonstrated the Thy1-ChR2 line marks a population whose activation is sufficient to enhance fear extinction (Jasnow *et. al*.^15^). Examination of differentially regulated genes identified using FACS and RNA sequencing reveals many genes that mark neuron populations that consistently overlap with the Thy1-eYFP populations. Finally, using drugs selectively targeting one identified marker, NTSR2, yields behavioural results that consistently recapitulate those observed with optogenetic and chemogenetic manipulations.

As is well known, use of the Thy1.2 expression cassette may generate mice with drastically different transgene expression patterns^16^. Similar expression patterns across mouse lines likely result from coincidental marking of a common developmental population originating from the pallial zones of the telencephalon^17^. Thus, we do not claim that Thy1 is a marker of only the amygdala Fear-Off population, but rather that these mouse lines conveniently mark a common developmental population generating a population of neurons, including the Fear-Off pyramidal neurons within the BLA in adulthood. Additional consideration must be given to the fact that within the Thy1-eNpHR mouse brain, populations in addition to the BLA population examined are labelled, specifically hippocampal and some cortical neurons. As there are many cell bodies and processes labelled in the BLA, it impossible to determine whether extrinsic terminals are also labelled, leaving the possibility open that some effects may be due to inhibition of terminals or fibres of passage. Behavioural replication using virally induced Gs-DREADD gives evidence that activity specifically of BLA Thy1 neurons is involved in fear learning; however, further study is necessary.

Highly specific micro-iontophoresis of muscimol specifically into the BLA blocks extinction consolidation without within-session effects^10^. In our hands, selective inhibition of Thy1 neurons appears to allow maintenance of activity of the previously silenced Fear-On circuitry. Thus, the fear circuit may be artificially unbalanced, and we observe enhanced within-session fear expression, in addition to previously observed deficits in extinction consolidation.

NTSR2, less studied than its high-affinity partner NTSR1, is a Gq-coupled signalling protein identified as a low-affinity NTSR with highly selective binding to levocabastine^44^. Recent reports from Tye and colleagues have demonstrated differential roles in behaviour for BLA neurons projecting to the CeA and NAc. The high-affinity NTSR1 may mark a CeA projecting population that supports fear expression^45^. NTSR1 was not found to be differentially regulated in Thy1-eYFP neurons in our study, suggesting a dynamic and potentially complementary role for neurotensinergic signalling in fear learning, perhaps dependent upon differential projection patterns^45^. It is possible these populations correspond to IL/PL projecting populations reported by Senn *et al*.^11^; however, further study is needed^11^. Thus, we propose that NTSR1 supports fear expression and NTSR2 supports fear inhibition via their differential expression within the BLA.

Results demonstrating the fear suppressing effects of beta-lactotensin are very encouraging; however, more research is necessary to clearly define the mechanism by which this compound is working. There have been reports of beta-lactotensin mediating anti-nociception, anxiolysis and fear memory modulation^42^,^46^,^47^,^48^. Although this is the first report examining intra-amygdala application of this compound in the context of auditory fear conditioning, future studies will need to rigorously dissect the mechanisms of this fear-suppression phenotype. Analysis of projection patterns of BLA Thy1-Cre neurons reveals a strong preference for regions commonly thought to be associated with appetitive learning and fear suppression such as the NAc, medial prefrontal cortex and mITC. Recently, there has been a great deal of discussion concerning the implications of a heterogeneous BLA population with distinct sets of neurons projecting to, for example, the CeA or the NAc depending on whether they convey information with negative or positive valence, respectively^45^. Here we suggest that Thy1 labelled neurons, acting as proxy for NTSR2-expressing neurons, correspond to the Fear-Off, and possibly positive valence neurons, based upon their role in behavioural and projection patterns.

## Methods

### Animals

Adult (8–12 week) B6.Cg-Tg(Thy1-eYFP)HJrs/J(Thy1-eYFP), B6.Cg-Tg(Thy1-COP4/EYFP)18Gfng/J (Thy1-ChR2-EYFP), FVB/N-Tg(Thy1-Cre)1Vln/J (Thy1-Cre), B6;SJL-Tg(Thy1-hop/EYFP)2Gfng/J (Thy1-eNpHR) and C57BL/6J mice were obtained from Jackson Laboratories (Bar Harbor, ME, USA). All mice were group housed and maintained on a 12:12 h light:dark cycle. Mice were housed in a temperature-controlled colony and given unrestricted access to food and water. All procedures performed conformed to National Institutes of Health guidelines and were approved by Emory University Institutional Animal Care and use Committee.

### Surgical procedures

Mice were deeply anaesthetized with ketamine/dexdormitor (medetomidine) mixture and heads fixed into stereotaxic instrument (Kopf Instruments). Stereotaxic coordinates were identified from Paxinos and Franklin (2004) and heads were leveled using lambda and bregma. To allow for optical inhibition (Fig. 3), mice were implanted unilaterally with an optical fibre (length 5 mm, 0.22 numerical aperture, 200 μm core; Thor labs) housed in a ceramic ferrule (Thor Labs) just dorsal to the BLA (−1.8 mm AP, ±3.4 mm ML, −4.8 mm DV) implants were randomized to side. Ferrules were adhered to the skull with adhesive, then a protective head cap was constructed using dental cement. For viral delivery (Fig. 4), a 10 μl microsyringe (Hamilton) was lowered to coordinates just above BLA and 0.5 μl of virus solution was infused at 0.1 μl min^−1^ using microsyringe pump. Virus solution contained either purified AAV_5_-hSyn-DIO-rM3D(Gs)-mCherry or AAV_5_-hSyn-eGFP in PBS (UNC Viral Vector Core). After infusion, syringes rested at injection for 15 min then slowly were withdrawn. After bilateral infusion, incisions were sutured closed using nylon monofilament (Ethicon). For pharmacological experiments (Fig. 7), mice were implanted bilaterally with a guide cannula (length 5 mm, 22 G, Plastics One) so that infuser tip rested just above the BLA as before. Guide cannulae were attached to the skull with adhesive and a head cap was constructed using dental cement. After dental cement cured dummy stilets were placed into cannulae. For all surgeries, body temperature was maintained using a heating pad. After completion of surgery, anaesthesia was reversed using Antisedan (atipamezole) and mice were allowed to recover on heating pads.

### Laser delivery

Optogenetic inhibition was achieved using a 50 mW DPSS 593 nm laser (Ikecool Inc., Anaheim, CA, USA). Laser-coupled fibre was attached to an optical fibre patch chord via a rotary joint (Doric) and suspended above behavioural testing chambers. Patch cords were attached directly to a chronically implanted optic fibre. Animals received 30 s of 10 mW (79.55 mW mm^−2^) tonic light during ‘tone on' epochs of cued fear behaviours. For open-field experiments mice received tonic light for alternating 2.5-min light-on but not light-off periods.

### Drug administration

CNO (Sigma) was diluted in sterile saline and administered at 1 mg kg^−1^ i.p. beta-lactotensin (National Institute of Mental Health (NIMH)) used for i.p. experiments (Fig. 7c) was prepared in sterile saline and administered at 30 mg kg^−1^ 2 h before behavioural testing. Beta-lactotensin used for initial intra-amygdala experiment (Fig. 7d) was prepared in 5% DMSO in sterile saline and administered at 90 μg perhemisphere 30 min before behavioural testing. Levocabastine (Sigma; Fig. 7d) was prepared fresh before the administration in 5% DMSO and administered at 1.5 μg per hemisphere. Beta-lactotensin used for follow-up replication (Fig. 7e) was prepared in sterile saline and administered at 30 μg per hemisphere 30 min before behavioural testing.

### Behavioural assays

*Auditory cue-dependent fear conditioning*. Mice were habituated to fear-conditioning chambers (Med Associates Inc., St Albans, VT) for 10 min each of 2 days before fear conditioning. Mice were conditioned to five tones (10 for Thy1-Cre experiment, Fig. 4; 30 s, 6 kHz, 65–70 db) co-terminating with a foot shock (1 s 0.4 mA(weak), or 0.65 mA(regular) depending on session).

*Auditory cue-dependent fear expression and extinction*. Cue-dependent fear extinction was tested 24 h after fear conditioning and extinction retention occurred 24 h after fear expression. For extinction, mice were placed in a novel context and exposed to 15 or 30, 30 s tones with an inter trial interval of 60 s. Freezing was measured using Freeze View software (Coulbourn Instruments Inc., Whitehall, PA, USA) or hand scored by two blinded experimenters (Fig. 3).

### Behavioural tests for c-fos expression experiments

As above, animals were habituated to training chamber. As above, animals were fear conditioned to 5 CS/US pairings. For fear extinction, mice were extinguished to 30 CS tones in an alternate context. The next day for fear extinction retention testing, mice were re-exposed to a single CS in the extinction context. Mice were killed as below 90 min following cessation of behaviour.

### Open field

Open-field chambers (Med Associates) were placed in a dimly lit room. Mice were placed in the chamber for 10 min and allowed to explore. For optogenetic experiments mice received light during the second and final 2.5 min of the session.

### Dissociation of amygdala tissue for FACS

For each of three replicates where RNA was collected from isolated neurons three groups of three Thy1-eYFP mice and several control C57 mice were rapidly decapitated and bilateral 1 mm amygdala punches were taken within 2 min. To minimize RNA degradation each solution used was prepared with RNAse Out (Life Technologies Inc., Bedford, MA, USA) and kept on ice. Each pair of amygdala punches was homogenized with a razorblade on a cold glass Petri dish then transferred to cold Hibernate A (Life Technologies). Tissue was spun down and supernatant replaced with 1 ml Accutase (Innovative Cell Technologies, Inc., San Diego, CA, USA) and allowed to digest for 30 min while rotating at 4 °C. Digested tissue was spun down and resuspended in cold Hibernate A. Three sets of punches were combined (for a total of *N*=3 mice per sample) and tissue was manually dissociated by trituration using fire polished Pasteur pipettes with diameters 1.3, 0.8 and 0.4 mm in series, where supernatant was collected and volume restored between each step. Cell suspensions were spun down and cell pellet resuspended in Hibernate A. Cell suspensions were filtered through pre-wetted 100 and 40 μm filters (BD Biosciences, Inc., San Jose, CA, USA) in series.

### Immunolabeling cell suspension for FACS

Cells were fixed by adding equal volumes of 100% ice cold EtOH and incubating for 15 min. Fixed cells were spun down and resuspended in sterile PBS. After removing appropriate aliquots for gating and compensation controls fixed cells were incubated with biotinylated anti-NeuN antibody (1:1,000, Milipore) and Hoechst (1:1,000, Sigma) while rotating at 4 °C. Cells were pelleted and washed with PBS. Cell suspensions were incubated with secondary allophycocyanin-labelled streptavidin (1:1,000, Invitrogen) for 30 min while rotating at 4 °C. Cells were pelleted and washed twice with cold PBS.

### Flow cytometry

A FACS Aria II (BD Biosciences) was used for cell sorting (Flow Cytometry Core at Yerkes National Primate Center). A portion of cells collected from wild-type mice was used to gate based on forward scatter (FSC) and side scatter (SSC) light scattering, and Hoechst fluorescence characteristics. Another portion of wild-type cells incubated exclusively in secondary antibody was used to set threshold for nonspecific binding of allophycocyanin–streptavadin binding and auto fluorescence in the 488 nm channel. Finally, stained cells collected from Thy1-eYFP mice were sorted and samples collected. For initial characterization, samples were collected in PBS and samples examined under fluorescent microscope to verify correct sorting. Thereafter, cells were sorted directly into lysis media of RNeasy Micro Kit (Qiagen) and kept at 4 °C. After sorting was completed RNA was isolated according to manufacturer's instructions including on-column DNase treatment. Samples were combined into single tube, and RNA quantity and quality were determined using Bioanalyzer pico chip (Agilent).

### Real-time PCR

RNA was reverse transcribed and amplified using SMARTer HV kit (Clonetech). Quantitative PCR was performed on complementary DNA with each sample run in triplicate technical replicates. Reactions contained 12 μl Taqman Gene Expression Master Mix (Applied Biosystems), 1 μl of each forward and reverse primer, 1 μl of 5 ng μl^−1^ complementary DNA and 6 μl water. Primers were proprietary fluorescein amidite (FAM)-labelled probes from Life Technologies. Quantification of qPCR was performed on Applied Biosystems 7500 Real-Time PCR System. Cycling parameters were 10 min at 95 °C, 40 cycles of amplification of 15 s at 95 °C and 60 at 60 °C, and a dissociation step of 15 s 95 °C, 60 s at 60 °C, 15 s 95 °C. Fold changes of YFP+ over YFP− groups were calculated as ΔΔCT values normalized to levels of actin B mRNA. Values presented as fold change ±s.e.m.

### Immunohistochemistry

For visualization of Decorin and RSPO2, Thy1-eYFP mice were perfused and brains were post fixed for 2 h using Zamboni Fixative. For all other visualizations mice were perfused and brains were post fixed for 2 h with 4% paraformaldehyde. Free-floating brain sections (35 μm) were rinsed in PBS, then in blocking solution (PBS, 10% normal goat serum, 0.25% Tween-20 and 0.4% Triton-X 100) for 1 h at room temperature and incubated for 24 h at 4 °C with the following antibodies in PBST: DKK3 (1:200, Abcam), TGFB2 (1:5,000, Abcam), Wnt7a(1:5,000, Santa Cruz), RSPO2 (1:200, Abcam), NTR2 (1:500, Santa Cruz) and Decorin (1:1,000, Santa Cruz). Sections stained for NTR2 underwent an amplification process, which consisted of a PBS rinse, 30 min incubation in peroxidase-labelled goat anti-rabbit (1:1,000, Vector Labs) at room temperature, another PBS rinse and 10 min in Fluorophore Tyramide Working Solution (TSA Plus Cyanine3 System)

All other sections were rinsed in PBS and incubated for 2 h at room temperature with either Alexa-Fluor 568 donkey anti-goat (1:1,000, Invitrogen) or Alexa-Fluor 594 goat anti-rabbit (1:1,000, Invitrogen) depending on the primary antibody's host. Following another PBS rinse, sections were mounted, then stained with Hoechst (1:1,000, Sigma) before being given a final PB rinse and cover-slipped with Mowiol mounting medium.

### RNA-seq library preparation

Libraries were generated from 1 ng of total RNA using the SMARTer HV kit (Clonetech), barcoding and sequencing primers were added using NexteraXT DNA kit. Libraries were validated by microelectrophoresis, quantified, pooled and clustered on Illumina TruSeq v3 flowcell. Clustered flowcell was sequenced on an Illumina HiSeq 1,000 in 100-base single-read reactions.

### Analysis of RNA sequencing data

RNA sequencing data was analysed using Tuxedo DESeq analysis software. Differential expression between YFP+ and YFP− groups were obtained and used for further analysis. Using the *q* value of <0.05 as a cutoff, only highly significant returns were used for further analysis. To ensure that genes had a large enough difference in expression to warrant pharmacological manipulation, only those with differences in expression greater than 2^0.5^ or ∼141% were considered. Next using the ‘Drug Gene Interaction Database' returns were examined for having a known pharmacological agent that modifies its activity. Genes lacking viable pharmacological modulators were eliminated.

### Statistics

Statistical analyses were performed using Prism 6 by Graph Pad. All data presented as mean±s.e.m. Differential c-fos expression was examined using a two-way ANOVA with behavioural condition as the between-subjects factor and Sidak's multiple comparisons test versus Home Cage. For comparison of eight c-fos expression groups, two-way ANOVA was performed followed by Sidak's *post hoc* multiple comparisons test. All behavioural experiments were examined using a repeated measures ANOVA with drug or optogenetic stimulation as the between-subjects factor and tone presentation as the within subject factor. Open-field activity (time in centre and distance travelled) was compared using a Student's *t*-test between carrier and non-carrier groups. For qPCR, ΔΔCT values of data were compared by Student's *t*-test between YFP+ and YFP− groups. For all tests statistical significance was set at *P*<0.05.

### Analysis of BLA Thy1-Cre projections

Three months post-viral infusion animals were killed and tissue was prepared as above. Slices were initially examined to confirm that cell bodies were labelled primarily in the BLA and that sight of expression conformed with target area. Slices were examined broadly with no *a priori* hypotheses about projection patterns. Images were taken using consistent exposure times within subject to preserve consistency of brightness of staining. Images at lower exposure time were taken of BLA for publication purposes to avoid over-exposure of YFP expressing cell bodies. Areas were characterized broadly as having either strong or weak fluorescent expression. From areas found to have strong expression, those known to play a role in fear learning and expression are highlighted in text.

### Data availability

The data that support the findings of this study are available from the corresponding author upon request

## Additional information

**How to cite this article:** McCullough, K. M. *et al*. Molecular characterization of Thy1 expressing fear-inhibiting neurons within the basolateral amygdala. *Nat. Commun.* **7,** 13149 doi: 10.1038/ncomms13149 (2016).

## Supplementary Material

Supplementary Information Supplementary Figures 1 - 15, Supplementary Discussion and Supplementary References
