# Supplementary material for: Molecular characterization of Thy1 expressing fear-inhibiting neurons within the basolateral amygdala
Source: Nat Commun. 2016 Oct 21;7:13149. doi: 10.1038/ncomms13149 (PMC5078744; doi:10.1038/ncomms13149)
Supplement: Supplementary Information — Supplementary Figures 1 - 15, Supplementary Discussion and Supplementary References [file ncomms13149-s1.pdf]

Supplementary Figures

Supplemental Figure 1

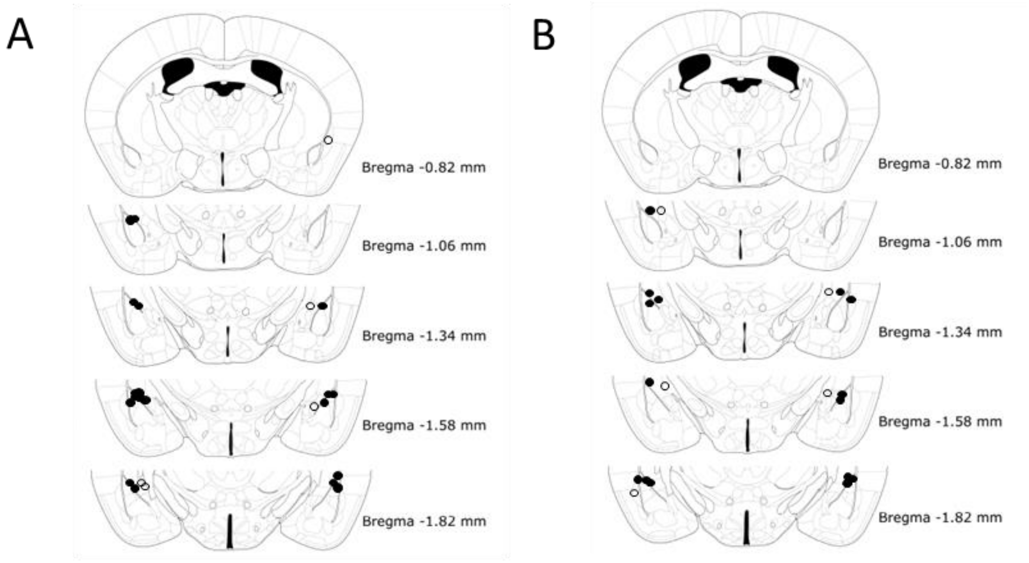

<u>*Supplementary Figure 1.* Schematic of fiber optic fiber tip placement.</u>
Schematic of locations of fiber optic tip location for **A.** inhibition with yellow laser light during fear acquisition and **B.** inhibition with yellow laser light during fear extinction sessions. Hits scored marked by filled circles and misses marked as open circles.

Supplemental Figure 2

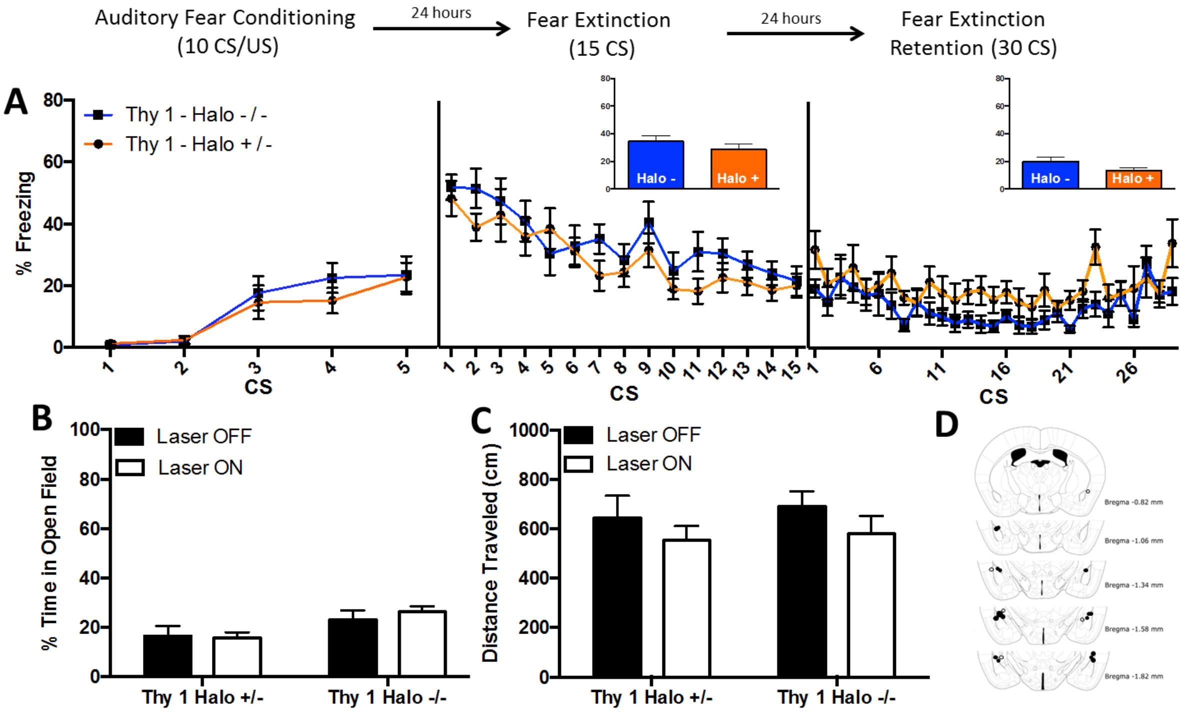

<u>*Supplementary Figure 2*. Genetic effects are not responsible for changes in fear expression of Thy1-eNpHR mice.</u>

A. Thy1-eNpHR carriers and non-carrier littermates that were cannulated and fear conditioned do not express any differences in fear behaviors in the absence of light stimulation. **B.** Mice exhibit no differences in anxiety like behavior as indicated by similar time spent in the center of an open field chamber regardless of genotype or status of laser (ON or OFF). **C.** Mice exhibit no differences distance traveled in open field chamber regardless of genotype or status of laser (ON or OFF). **D.** Schematic of fiber optic tip placement. Hits scored marked by filled circles and misses marked as open circles.

Supplemental Figure 3

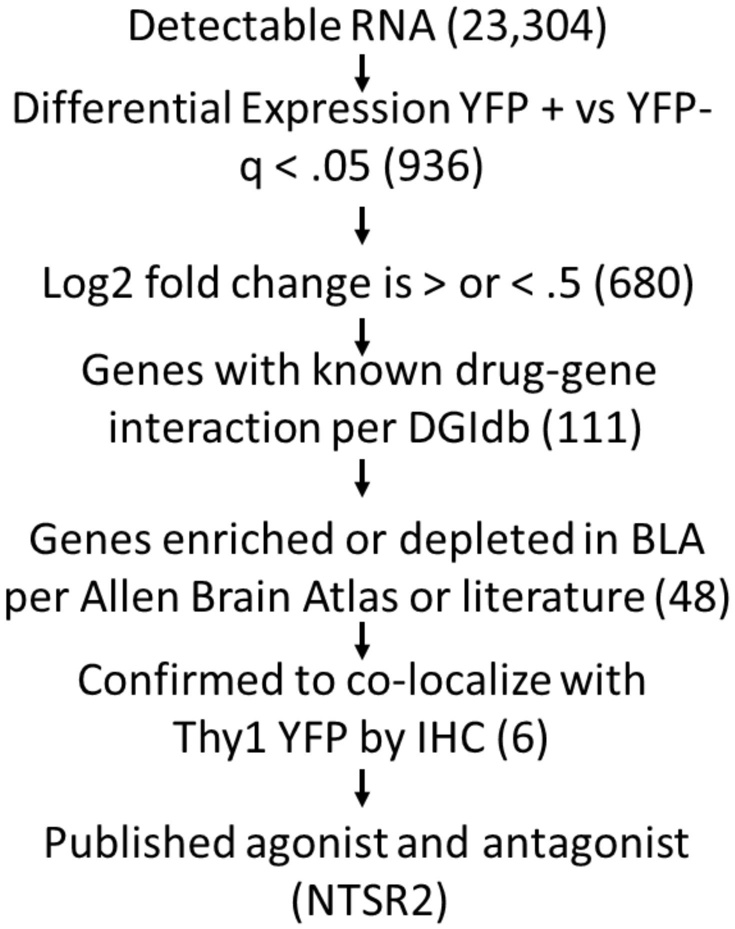

Supplementary Figure 3. Flow chart of strategy for analysis of RNA sequencing differential expression data. First, only highly significant genes were taken so that any difference score with q>.05 was discarded. Next, only genes whose expression differed from control by more than 2^.5 were taken. Genes of interest were entered into the DGIdb drug-gene interaction tool and any gene without a pharmacological modulator was discarded. Genes on resulting list were examined for expression patterns on the Allen Brain Atlas or in the published literature. Those with visibly enriched expression in one or more amygdalar nuclei were selected. Finally based upon the previous two criteria, genes were chosen for protein analysis with immunohistochemistry. NTSR2 was selected for pharmacological analysis based upon published reports of both an agonist and antagonist.

Supplemental Figure 4

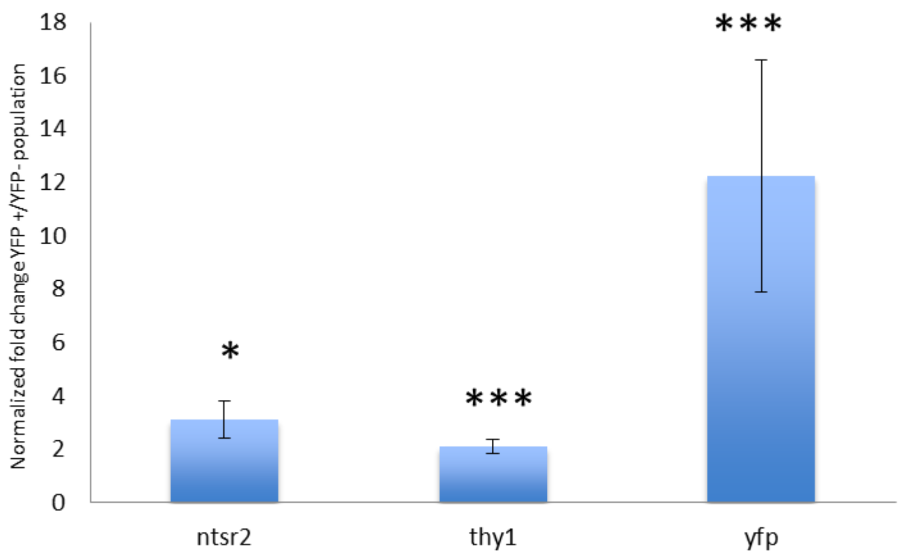

Supplementary Figure 4. Replication of RNA sequencing results with qPCR.
Amplified cDNA generated from RNA taken from FACS sorted neurons was analyzed with qPCR. Resulting fold changes of gene expression in RNA taken from YFP positive neurons vs. YFP negative neurons are represented in bar graph. In all panels Error Bars indicate mean +/- SEM.

Supplemental Figure 5

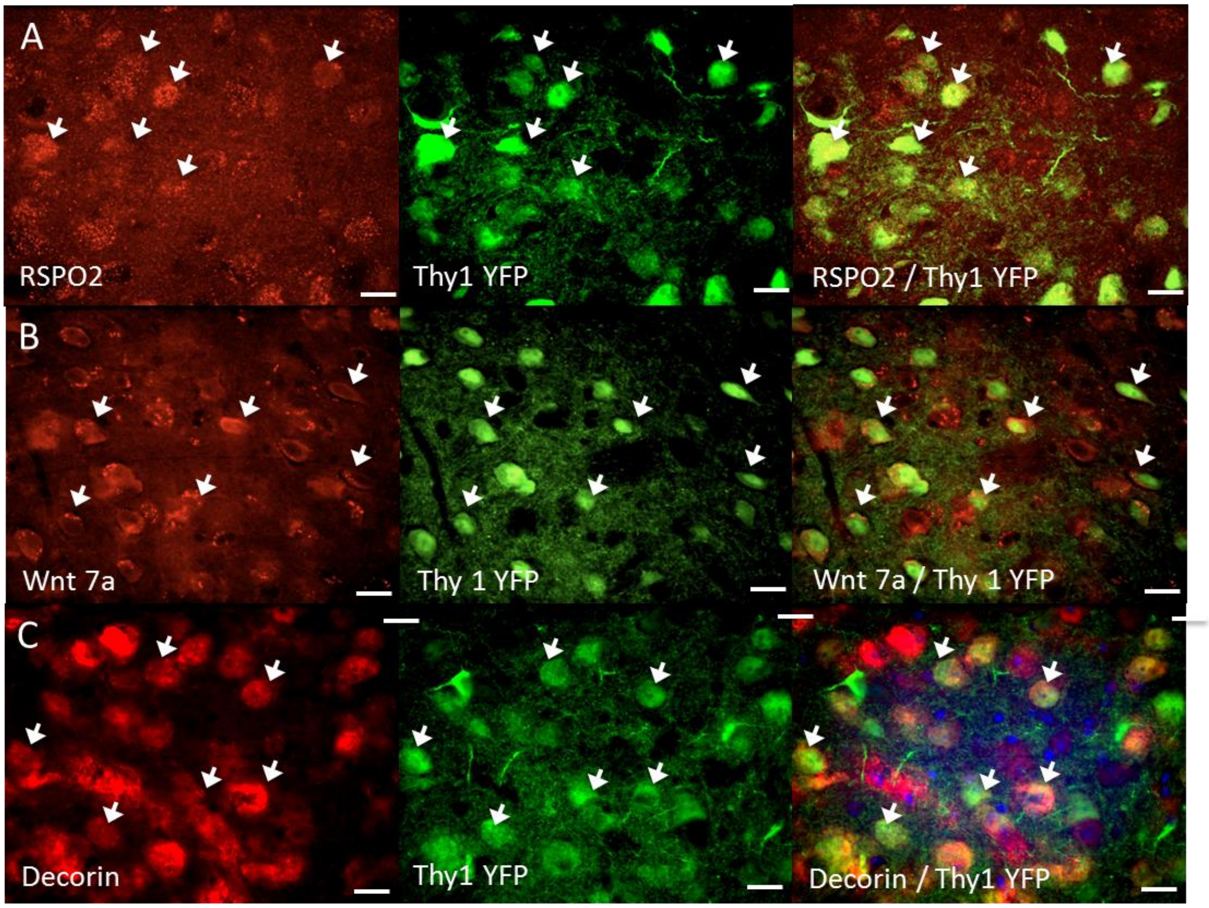

Supplementary Figure 5. Co-localization of Thy1-eYFP with additional differentially expressed genes.

Molecular characterization of Thy1-eYFP expressing neurons of the basolateral amygdala was completed on tissue from Thy1-eYFP line H mice. Coronal sections were stained for protein of interested using immunohistochemistry visualized using secondary antibodies emitting in the red spectrum. Thy1-eYFP strongly co-localizes with **A.** RSPO2, **B.** Wnt 7a, and **C.** Decorin. Images were captured using a confocal microscope. Scale Bar = 20 um.

Supplemental Figure 6

A

	YFP + DAPI	DAPI alone
Thy 1 YFP vs. DAPI	17.1	83.5

B

Protein of Interest	P+ / YFP-	P+ /YFP +	P- / YFP+
TGFB2	9.8	15.6	.2
Dkk3	4.3	16.8	0
Wnt 7a	6.6	16.4	0
NTSR2	3.6	17.3	0
RSPO2	3.8	17.1	0.1
Decorin	4.7	18.8	0.2

Counts of expression presented as raw averages of counts per field examined.

C

	YFP +
Thy 1 YFP vs. DAPI	100%

Protein of Interest	P+ / YFP-	P+ /YFP +	P- / YFP+
TGFB2	38.3%	60.9%	0.7%
Dkk3	20.4%	79.6%	0%
Wnt 7a	28.7%	71.3%	0%
NTSR2	17.2%	82.8%	0%
RSPO2	18.1%	81.4%	0.4%
Decorin	19.8%	79.3%	0.8%

Counts of expression presented as a percentage of total fluorescent positive cells counted (X/((P+) + (YFP+))).

Supplementary Figure 6. Quantification of co-localization between Thy1-eYFP and additional proteins of interest.

Immunoreactivity was analyzed in a volumetric manner using confocal microcopy where neurons intersecting with the proximal Z-plane and two boarders were excluded. **A.** Initially, to determine the percentage of total cells that express YFP, Thy1-YFP slices were stained with DAPI alone. Approximately 20% of total cells in BLA regions examined express YFP. **B.** Next, after IHC cells expressing either green (YFP+) or red (Protein of interest + (P+)) fluorescence were counted as single positive respectively (P+ / YFP- or P- / YFP+) while cells expressing both red and green fluorescence were counted as double positive(P+/ YFP +). Single positive Thy1-eYFP and gene of interest neurons were counted as well as double positive neurons. Counts represent the average number of fluorescent neurons counted per stack (n=15). These counts demonstrate that almost 100% of all YFP+ cells counted are also stained with the protein of interest. Interestingly the number of cells expressing the protein of interest but not YFP varies considerably (between 38.3% to 17.2% of total number counted, see C) suggesting that the Thy1-YFP population may not be housed within a single homogeneous larger population, or that the level of detectable protein expression with IHC under-reports the cell population expressing mRNA for the gene of interest. **C.** Data presented in B re-represented as counts of expression as a percentage of total fluorescent positive cells counted (X/((P+) + (YFP+))).

Supplemental Figure 7

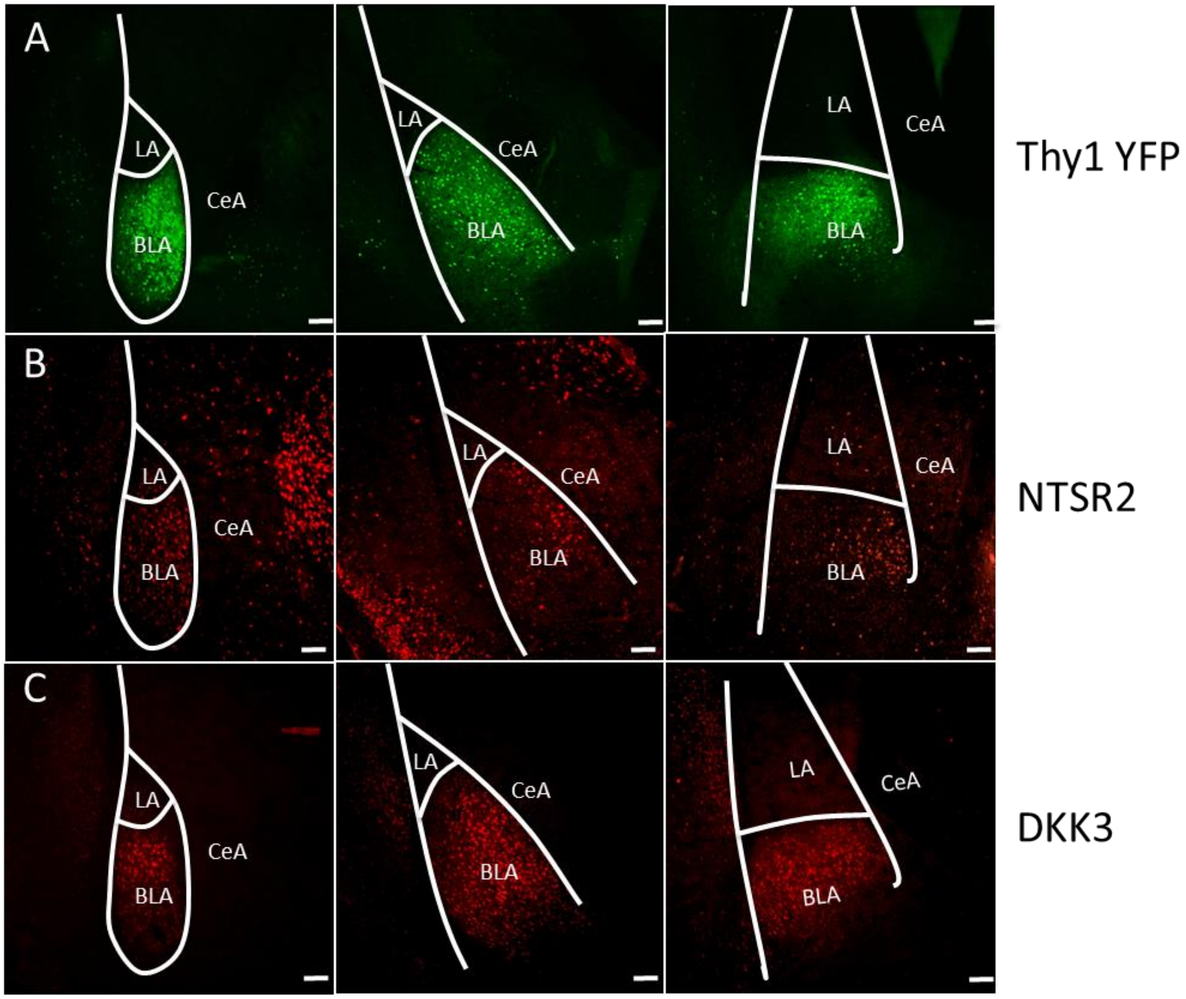

Supplementary Figure 7. Regional similarities in Thy1-eYFP, NTSR2, and DKK3 expression. Images were captured at lower magnification across the anterior-posterior axis of the amygdala. Both **B.** NTSR2 and **C.** DKK3 have similar expression patterns to Thy1-eYFP across the length of the amygdala. Scale Bar = 100um.

Supplemental Figure 8

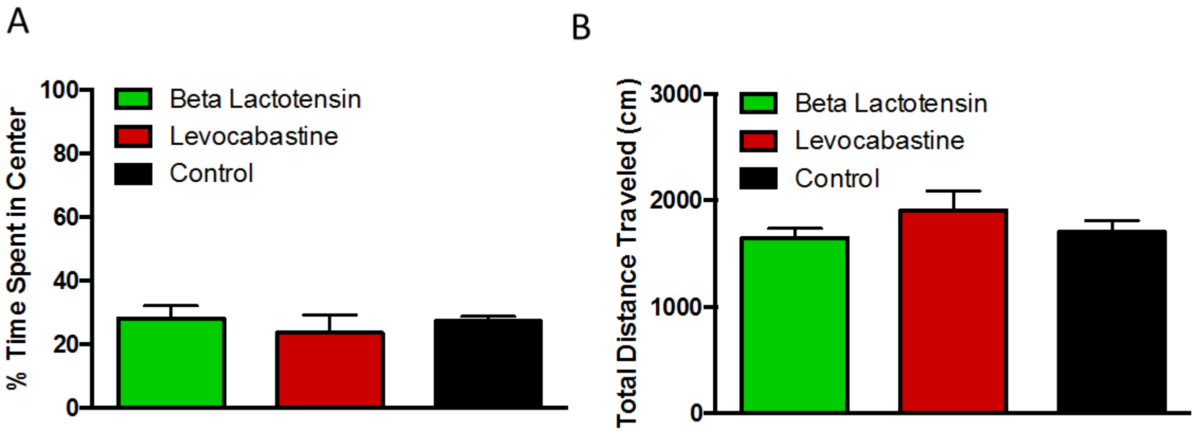

Supplementary Figure 8. Differences in fear behavior after drug delivery are not due to anxiety like behavior after drug administration.

Mice infused with Beta-Lactotensin, Levocabastine or vehicle 30 minutes before being placed in Open-Field box for 10 minutes express no differences in **A.** time spent in center or **B.** total distance traveled throughout 10 minute session were detected. In all panels Error Bars indicate mean +/- SEM.

Supplemental Figure 9

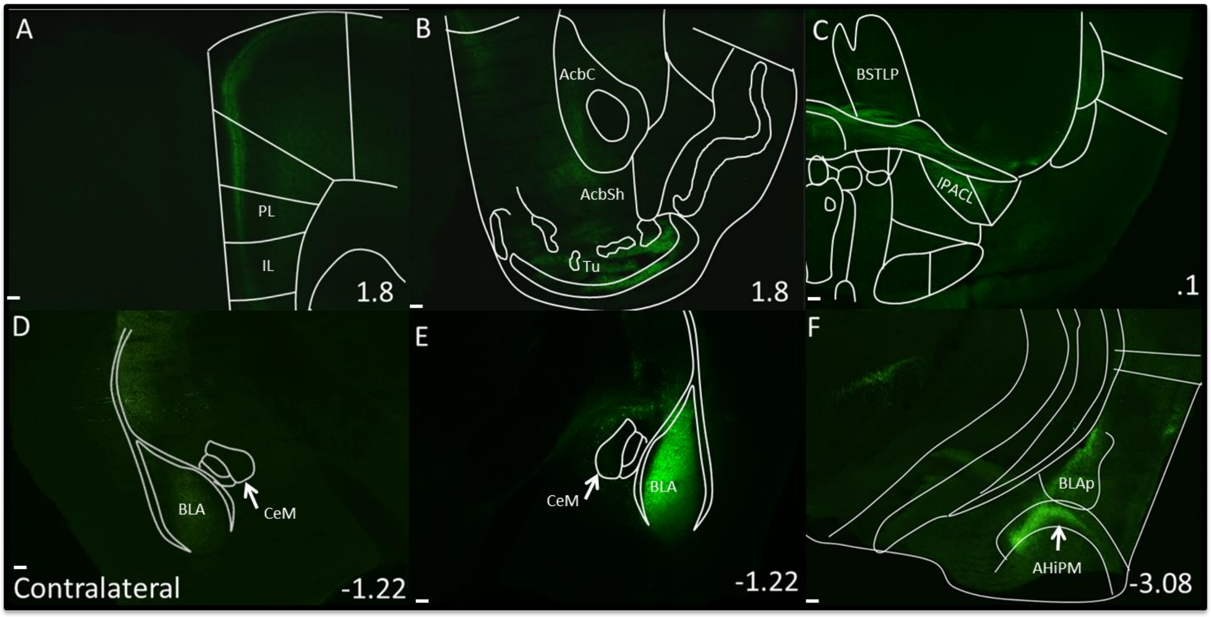

Supplementary Figure 9. Infusion of AAV-DIO-YFP into Anterior BLA Thy1-Cre mouse. Infusions of AAV-DIO-YFP centered on the anterior aspect of the BLA (-1.0 A/P) label populations that have moderate projections to the superficial layers of the PFC **A.** and the NAc **B**. Also observed are moderate projections to elements of the bed nucleus of the stria teminalis (BNST), the claustrum and the anterior insula (AI)). Additional fluorescence can be found in the contralateral BLA (**D**) and caudally in the posterior medial amygdalohippocampal area (**F**). Cell bodies are labeled around infusion site **E**. All Scale Bar = 100 um.

Supplemental Figure 10

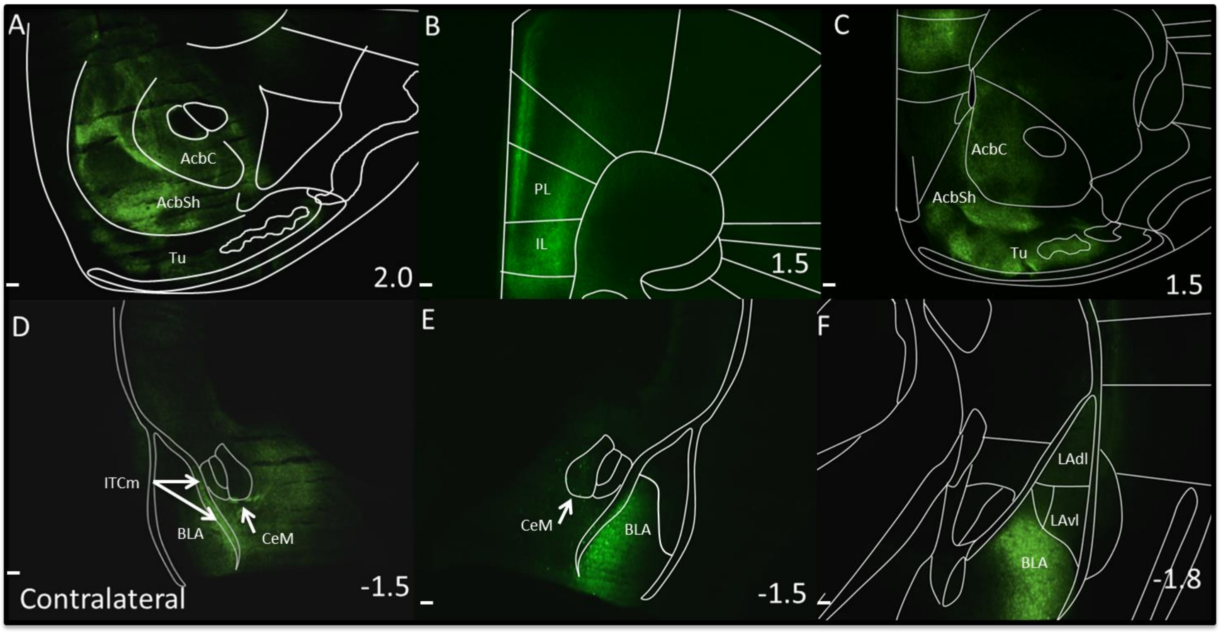

Supplementary Figure 10. Infusion of AAV-DIO-YFP into BLA Thy1-Cre mouse.

Infusions of AAV-DIO-YFP at -1.5 A/P label populations that project very strongly to the NAc **A** and **C**. Projections the superficial layers of the PFC remain although there are additional projections to the deeper layers of the vmPFC **B**. Interestingly, these infusions appear to mark neurons that project very strongly to the contralateral ITC, BLA and MeA**D**. Projections from marked neurons project strongly to several regions of the BNST**G**. Cell bodies are labeled around infusion site **E.** and **F**. All Scale Bar = 100 um.

Supplemental Figure 11

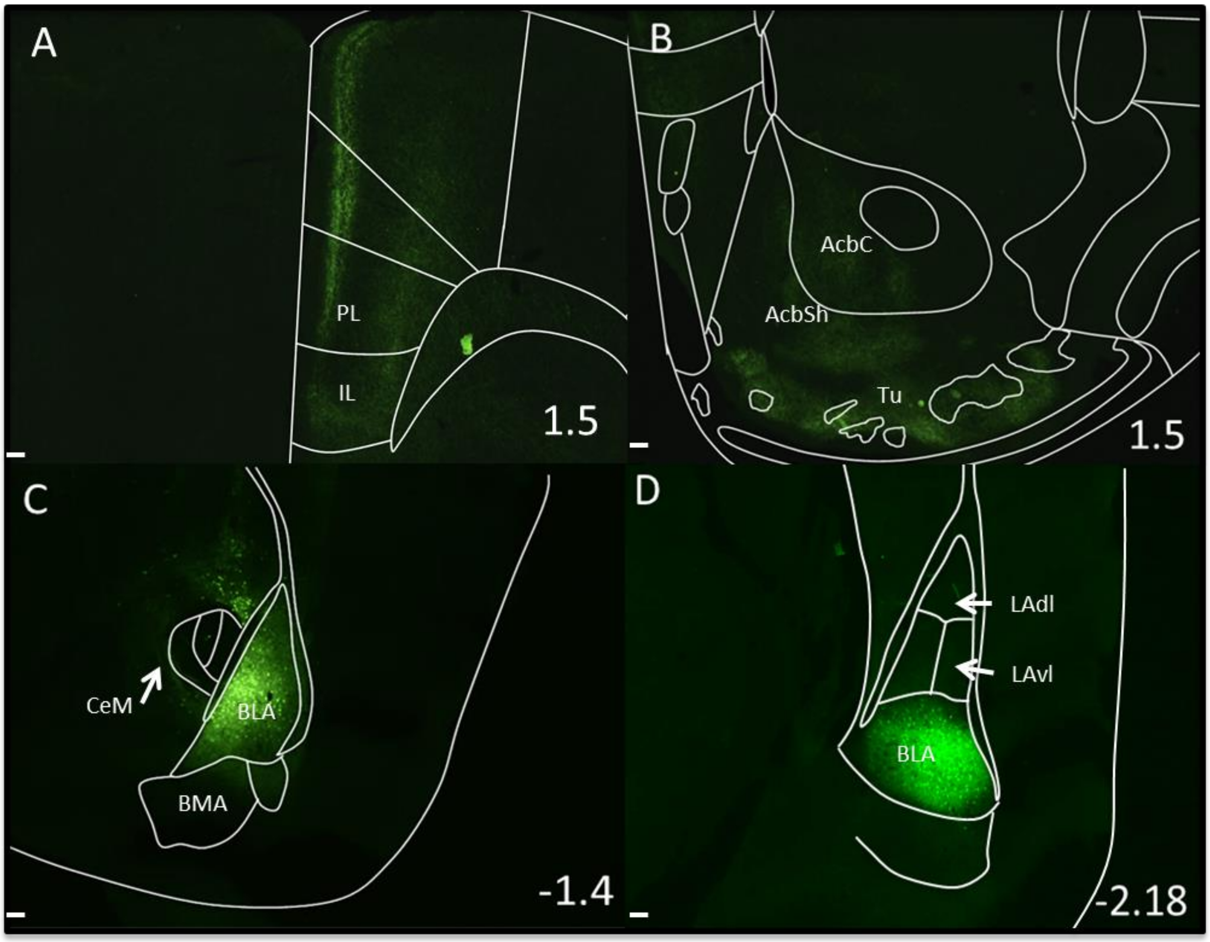

<u>*Supplementary Figure 11*. Infusion of AAV-DIO-YFP into Posterior BLA Thy1-Cre mouse.</u>
Infusions of AAV-DIO-YFP into the caudal aspect of the BLA (-2.0 A/P) label populations that project to the superficial layers of the PFC as well as to the deeper layers of the vmPFC **A**. Labeled neurons additionally project to the NAc **B**. Cell bodies are labeled around infusion site (**C** and **D**). All Scale Bar = 100 um.

Supplemental Figure 12

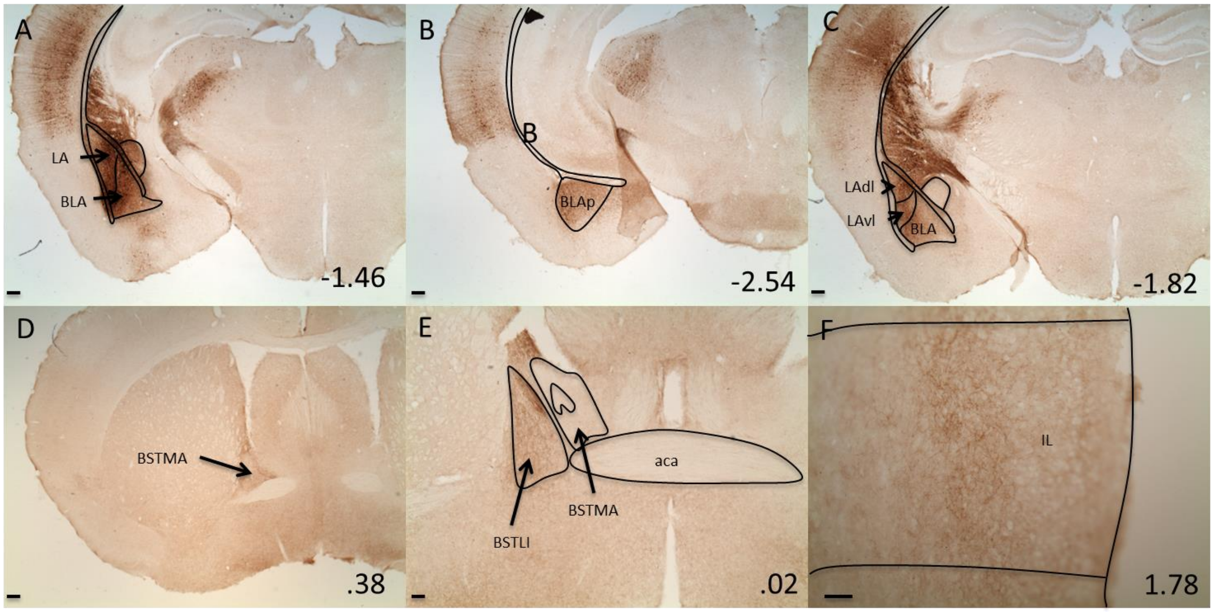

Supplementary Figure 12. Infusion of BDA into Anterior BLA.
Infusions of BDA (light field) into anterior aspect of BLA (-1.5 A/P) label populations with strong projections to posterior elements of the BLP. Infusion site **A.** and **C**. Some projections can be found in some nuclei of BNST **D.** and **E**. Moderate numbers of projections are observed in infralimbic cortex. A-D Scale Bar = 200 um. E Scale Bar = 100 um. F Scale bar =40 um.

Supplemental Figure 13

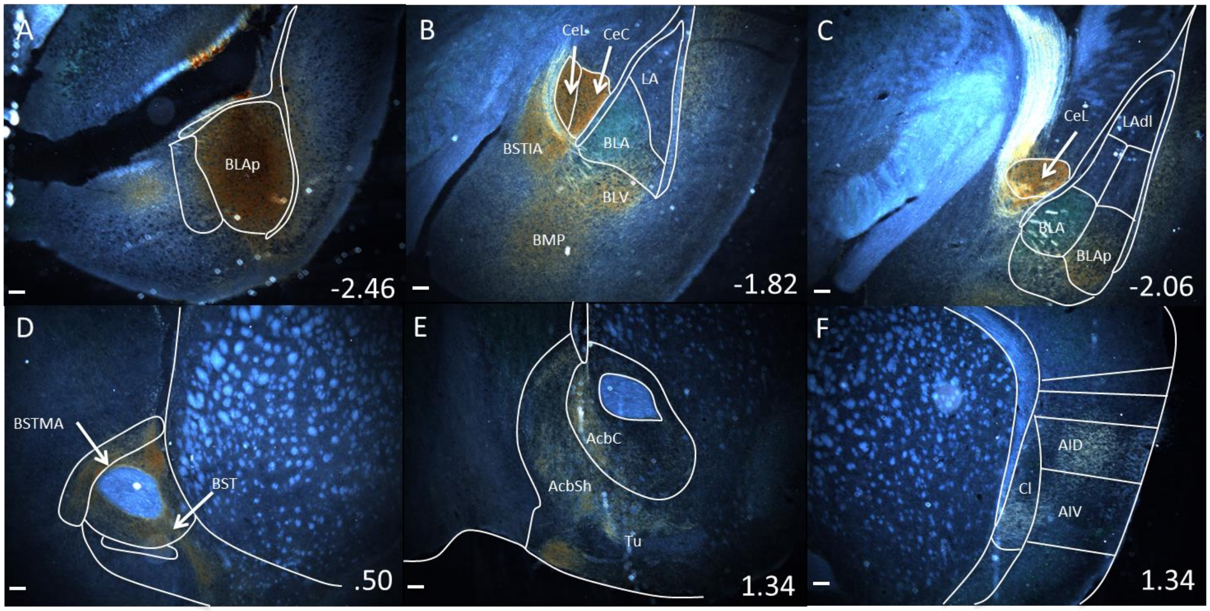

Supplementary Figure 13. Infusion of BDA into Posterior BLA

Infusion of BDA (dark field) into posterior aspect of BLA (-2.5). Site of infusion (**A**). BDA labels a population with strong projections to the CeA (**B** and **C**). Strong projections to the BNST (**D**), NAc (**E**) and anterior insula (**F**) are also observed. All Scale Bars = 100um.

Supplemental Figure 14

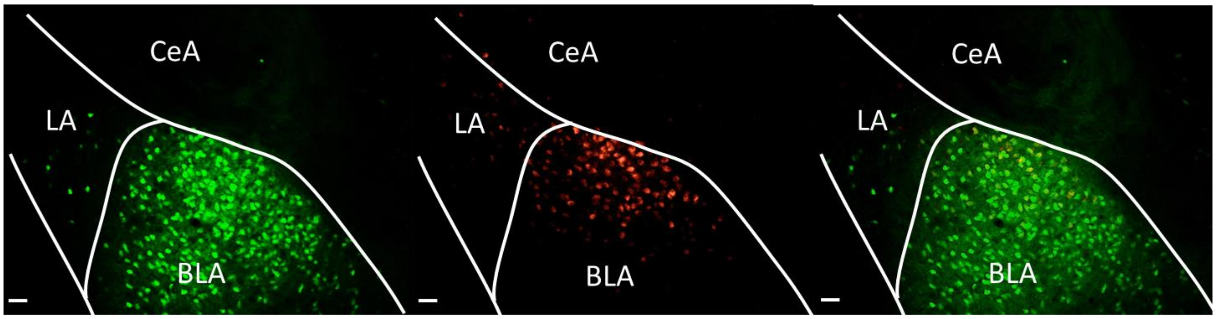

Supplementary Figure 14. Regional specificity of cre-recombinase mediated mCherry expression.

Cre-dependent mCherry expression resulting from infusion of AAV-EF1a-DIO-mCherry into Thy1-cre mouse. mCherry expression is observed strongly in BLA as well as weakly in the LA and a Paracapsular region of the CeA. All Scale Bars = 50 um.

Supplemental Figure 15

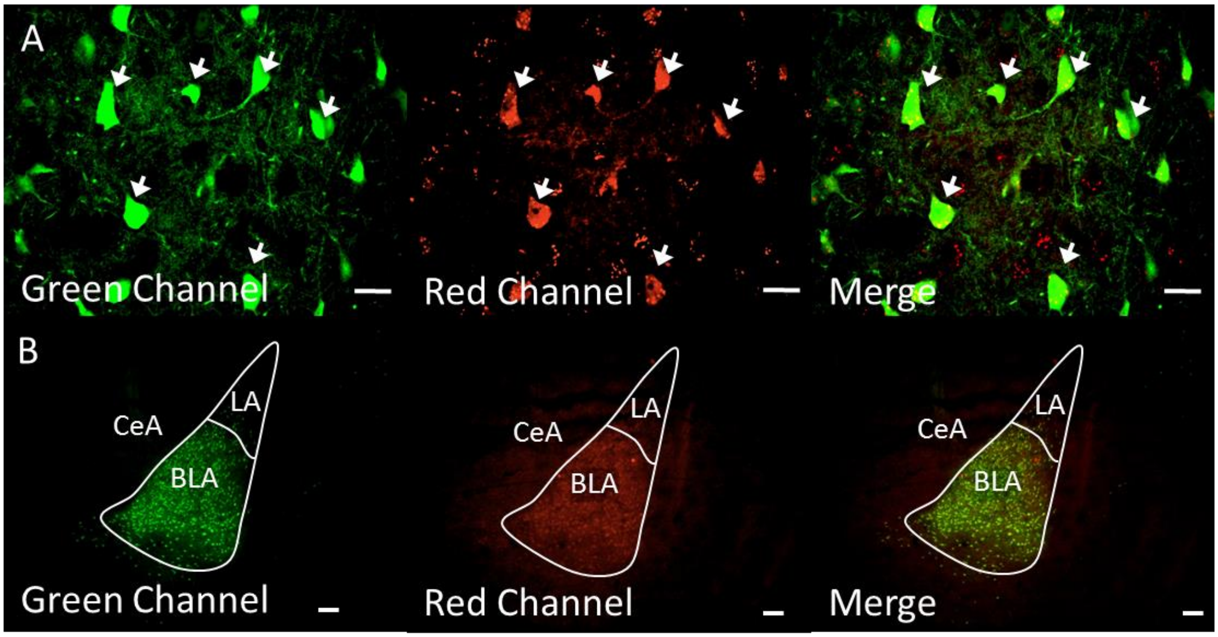

<u>*Supplementary Figure 15*. Double transgenic Thy1-eYFP/ Thy1-Cre mice have red-shifted expression in Thy1-eYFP neurons.</u>
Examination of tissue from double transgenic mice reveals the presence of a red-emitting fluorophore that completely overlaps with Thy1-eYFP expression at a **A.** cellular level and **B.** regional level. This expression is detected in all Thy1-eYFP neurons throughout the brain. A. Scale Bars = 20 um, B Scale Bar = 100 um.

Supplementary Discussion

Across mouse lines, the Thy1 expression cassette causes transgene expression in convergent populations of neurons. It is important to acknowledge that the generation of Thy1 transgenic lines by insertion of the Thy1.2 expression cassette can yield mice with drastically different transgene expression patterns [1]. The most well characterized Thy1-eYFP line, line-H, has strong expression in layer 5/6 cortical neurons as well as hippocampal and amygdala populations [2]. It is likely that similar expression patterns across mouse lines result from coincidental marking of a common developmental population originating from the pallial zones of the telencephalon[2]. Thus, we do not claim that Thy1 is a marker of the amygdala Fear-Off population, but rather that these mouse lines conveniently mark a common developmental population generating a population of neurons including the Fear-Off pyramidal neurons within the BLA in adulthood. Previously, using ISH and IHC we have demonstrated that both the Thy1-ChR2 and Thy1-eYFP lines mark a subset of CaMKII expressing excitatory neurons (Jasnow et al., (2013)). These lines do not mark the entire excitatory population of the BLA; however, a large proportion of the total population is marked.

Jasnow and colleagues found that activation of BLA Thy1 neurons was sufficient to suppress excitatory transmission generated by electrical stimulation of the LA. We believe that BLA Thy1 neurons project to the medial ITC's, which provide strong feed-forward inhibition to the CeA; however, in this system it is difficult to visualize these clusters as they are located within the internal capsule. However, based upon supporting literature we understand that BA projections to mITC's are implicated in providing feed-forward inhibition to the CeM[3]. Further examination of the source of this inhibition in necessary.

An important consideration in this data is that optical inhibition was completed unilaterally. There is convincing data using optogenetics, inactivation and lesioning in multiple brain regions including amygdala, prefrontal cortex and auditory cortex to support that unilateral silencing is sufficient in many cases to examine the necessity of a cell population in behavior[4-6]. Additionally, bilateral DREADD manipulations confirm consistent roles for this Thy1-BLA population in fear behavior across optogenetic and chemogenetic modes of interrogation.

Characterization of Thy1-Cre expression patterns using post-natal Cre-recombinase dependent reporter viruses revealed expression patterns consistent with other lines. However, limited expression in the BLA of Thy1-Cre mice contrasts with the original characterization of this line and our own characterizations using developmentally available Cre-recombinase dependent reporter lines (*data not shown*). These expression patterns suggests that the Thy1 cassette is expressed much more promiscuously in the Thy1-Cre line during development, but takes on a more constrained expression pattern during adulthood. The use of reporter viruses to characterize Cre-recombinase expression provides further evidence to support the above observation. AAV-hSyn-DIO-rM3D(Gs)-mCherry and AAV-EIF1-DIO-mCherry were each infused separately into the BLA of Thy1-Cre or Thy1-eYFP/Thy1-Cre mice respectively. The resulting expression from an hSyn promoter was primarily constrained to the BLA. EF1a promoter virus produces strong BLA expression as well as weaker expression in a medial LA population and a small population in the capsular region of the CeA (Supplementary Figure 14). It is likely that the Thy1.2 cassette is able to drive some basal expression in most neurons, but surrounding control regions limit significant expression to the previously discussed developmental population. Expression patterns detected in Thy1-Cre mice indicate that the Thy1 promoter drives Cre-Recombinase expression primarily in the described BLA pattern with some minimal expression in other neuron populations. As a single molecule of Cre-Recombinase may be sufficient to drive recombination, depending on the sensitivity of the viral construct, different expression patterns of fluorescent marker are revealed in the Thy1-

Cre mouse. These observations, taken with convergent behavioral data across four Thy1 transgenic lines, suggests that Thy1 lines used in the present study mark a common regional population that contains fear inhibition circuitry. With regards to examination of projections originating from BLA Thy1 neurons, the data is purely descriptive. An in-depth quantified analysis of these projections would be of great value.

On a technical note, crossing the Thy1-eYFP and Thy1-Cre mouse lines results in the appearance of a weak red fluorescent signal in all Thy1-eYFP labeled cells, even in the absence of red fluorescent reporter virus (Supplementary Figure 15). This is easily distinguishable from transgenic mCherry expression described above, as it is quite weak and is found in Thy1-eYFP neurons throughout the brain. This signal likely results from a red shifting of a small percentage of transgenically expressed YFP. The cause of this red-shifting in unknown, but may result from a change in the intracellular conditions caused by the additive cellular stress of expressing two transgenes at high levels [7].

Examination of c-fos expression after fear behaviors demonstrates that Thy1-eYFP labeled neurons are recruited specifically during fear extinction expression whereas unlabeled cells are recruited preferentially during fear expression. The necessity of these neurons is examined by using a Thy1-NpHR mouse to optogenetically silence Thy1 neurons during behavior. Inhibition of labeled Thy1 neurons during CS presentations within the fear conditioning session leads to enhanced fear consolidation as measured the next day during a fear extinction test. Silencing Thy1 neurons during the fear extinction session leads to within-session increases in fear expression as well as blunted fear extinction consolidation the next day during the unstimulated fear expression test. This is in contrast to reports that muscimol administration, pharmacologically inhibiting the basal amygdala and BMA, prior to training has no effect on behavior and prior to extinction prevents fear expression[8]. However, more limited micro-iontophoresis of muscimol specifically into BLA does not produce within session effects, but does blunt fear extinction consolidation [9]. Selective inhibition of Thy1 neurons appears to allow maintenance of activity of the previously silenced Fear-On circuitry. Thus, the fear circuit may be artificially unbalanced, and we observe enhanced within-session fear expression in addition to previously observed deficits in extinction consolidation. Optical inhibition is unilateral, thus we do not see complete lack of extinction consolidation, as the contralateral amygdala is fully functional. It is important to highlight the temporal specificity of this approach, where Thy1 neurons are silenced only during CS presentation, suggesting that it is specifically the association between the CS and US that is being over-expressed and over-consolidated.

The use of cell-type specific whole genome expression analysis allows in-depth interrogation of the molecular identity of a neural population of interest [10,11]. Thy1-eYFP neurons were dissociated and sorted based upon their expression of a neuronal marker, NeuN, and Thy1 driven YFP. This allowed for the isolation of high quality RNA from a large number of Thy1-eYFP (YFP+, NeuN+) and other (YFP-, NeuN+) neurons. Of all cell bodies interrogated, 40% were NeuN positive while only ~2% were NeuN and YFP double positive. This approach has the advantage that the RNA sequencing data represents the average RNA content of Thy1-eYFP cells across the anterior-posterior axis of the amygdala as 8000-12,000 cells are isolated for each sample. However, because tissue punches likely contain cells from the CeA, LA, and BMA this method lacks the sensitivity to identify many transcripts specifically down-regulated in non-Thy1 neurons of the BLA that may have divergent functional roles. Furthermore by homogenizing non-Thy1-eYFP neurons into a single group this method washes out many differences between Thy1 neurons and other specific nuclei of the amygdala.

RNA sequencing yielded hundreds of transcripts that are differentially regulated between the Thy1-eYFP and other amygdala neurons. These were prioritized based upon a workflow designed to identify transcripts specifically upregulated in BLA Thy1 neurons that have previously been associated with pharmacological modulators (Supplementary Figure 3). Of those examined for protein expression patterns, *Tgfb2*[12] and *Dcn*[13] have complex interactions with TGF-Beta signaling, cell cycle and axon growth; *Wnt7a* [14], *Dkk3* [15], and *Rspo*-2 [16] regulate wnt/Beta-catenin signaling previously associated with fear modulation[17], as well as synaptic remodeling and plasticity, and possibly bipolar disorder; and *Ntsr2* [18] has complex signaling roles that influence the neuroendocrine and dopamine systems.

Examination of the protein products of these genes using immunohistochemistry revealed consistent regional overlap with Thy1 neurons. Importantly, although all genes had strong expression within the BLA, the extra amygdala expression patterns varied widely, suggesting that overlapping expression is a feature unique to BLA neurons. When investigated at a cellular level, all proteins examined had almost complete overlap with Thy1-eYFP expressing neurons although all marked some non-YFP expressing cells as well, suggesting that Thy1-eYFP marks a sub-set of these neurons. Quantification of co-localization was performed so that a 20μm thick slice was analyzed and labeled cells intersecting top (Z-axis) plane and two sides of the image were not counted (Supplementary Figure 6). Tissue used in DREADD experiments was stained for DKK3 and TGFB2 demonstrating that Thy1-Cre neurons similarly co-localize with these markers. Overall, immunolabeling suggests expression diversity within BLA neurons representing what may amount to a hierarchical system that delineates functionally divergent sub-populations. Importantly, when colocalization was examined with Thy1-eYFP neuron images taken in areas of strong YFP expression, many genes maintained expression outside the strict BLA pattern seen in Thy1-eYFP; therefore, counts of colocalization only apply to the BLA.

Data presented here demonstrate functional and molecular characterization of the BLA Thy1 population and identifies NTSR2 as a possible functional marker of a BLA Fear-Off population. Across several Thy1 transgenic lines, strong overlap in regional and cellular expression was observed. Manipulation using optogenetics and chemogenetics confirmed a consistent functional role in behavior suggesting that the Thy1 labeled neurons contain a BLA Fear-Off population. Genetic tracing reveals projection patterns to NAc, mPFC and ITCm, avoiding CeA, consistent with a Fear-Off / positive valence circuit. Isolation and RNA profiling of Thy1-eYFP neurons revealed a number of candidate genes that are upregulated in Thy1 neurons. Neurotensin Receptor 2 is strongly expressed in all Thy1-eYFP neurons and pharmacological manipulation using agonists or antagonists is able to enhance or suppress freezing respectively. These findings confirm that NTSR2, like Thy1, labels a population of the BLA containing functional Fear-Off circuitry, and activating the NTSR2 population may provide a novel approach to the clinical reduction of fear and enhancement of fear extinction.

Supplementary References

1 Feng, G. *et al.* Imaging neuronal subsets in transgenic mice expressing multiple spectral variants of GFP. *Neuron* **28**, 41-51 (2000).

2 Porrero, C., Rubio-Garrido, P., Avendano, C. & Clasca, F. Mapping of fluorescent protein-expressing neurons and axon pathways in adult and developing Thy1-eYFP-H transgenic mice. *Brain research* **1345**, 59-72, doi:10.1016/j.brainres.2010.05.061 (2010).

3 Busti, D. *et al.* Different fear states engage distinct networks within the intercalated cell clusters of the amygdala. *The Journal of neuroscience : the official journal of the Society for Neuroscience* **31**, 5131-5144, doi:10.1523/jneurosci.6100-10.2011 (2011).

4 Nomura, H. *et al.* Memory formation and retrieval of neuronal silencing in the auditory cortex. *Proceedings of the National Academy of Sciences of the United States of America* **112**, 9740-9744, doi:10.1073/pnas.1500869112 (2015).

5 Kim, H. S., Cho, H. Y., Augustine, G. J. & Han, J. H. Selective Control of Fear Expression by Optogenetic Manipulation of Infralimbic Cortex after Extinction. *Neuropsychopharmacology : official publication of the American College of Neuropsychopharmacology* **41**, 1261-1273, doi:10.1038/npp.2015.276 (2016).

6 LaBar, K. S. & LeDoux, J. E. Partial disruption of fear conditioning in rats with unilateral amygdala damage: correspondence with unilateral temporal lobectomy in humans. *Behavioral neuroscience* **110**, 991-997 (1996).

7 Elowitz, M. B., Surette, M. G., Wolf, P. E., Stock, J. & Leibler, S. Photoactivation turns green fluorescent protein red. *Current biology : CB* **7**, 809-812 (1997).

8 Sierra-Mercado, D., Padilla-Coreano, N. & Quirk, G. J. Dissociable roles of prelimbic and infralimbic cortices, ventral hippocampus, and basolateral amygdala in the expression and extinction of conditioned fear. *Neuropsychopharmacology : official publication of the American College of Neuropsychopharmacology* **36**, 529-538, doi:10.1038/npp.2010.184 (2011).

9 Herry, C. *et al.* Switching on and off fear by distinct neuronal circuits. *Nature* **454**, 600-606, doi:10.1038/nature07166 (2008).

10 Guez-Barber, D. *et al.* FACS purification of immunolabeled cell types from adult rat brain. *J Neurosci Methods* **203**, 10-18, doi:10.1016/j.jneumeth.2011.08.045 (2012).

11 Guez-Barber, D. *et al.* FACS identifies unique cocaine-induced gene regulation in selectively activated adult striatal neurons. *The Journal of neuroscience : the official journal of the Society for Neuroscience* **31**, 4251-4259, doi:10.1523/JNEUROSCI.6195-10.2011 (2011).

12 Fukushima, T., Liu, R. Y. & Byrne, J. H. Transforming growth factor-beta2 modulates synaptic efficacy and plasticity and induces phosphorylation of CREB in hippocampal neurons. *Hippocampus* **17**, 5-9, doi:10.1002/hipo.20243 (2007).

13 Esmaeili, M., Berry, M., Logan, A. & Ahmed, Z. Decorin treatment of spinal cord injury. *Neural regeneration research* **9**, 1653-1656, doi:10.4103/1673-5374.141797 (2014).

14 Fernando, C. V. *et al.* Diverse roles for Wnt7a in ventral midbrain neurogenesis and dopaminergic axon morphogenesis. *Stem cells and development* **23**, 1991-2003, doi:10.1089/scd.2014.0166 (2014).

15 Diep, D. B., Hoen, N., Backman, M., Machon, O. & Krauss, S. Characterisation of the Wnt antagonists and their response to conditionally activated Wnt signalling in the developing mouse forebrain. *Brain research. Developmental brain research* **153**, 261-270, doi:10.1016/j.devbrainres.2004.09.008 (2004).
16 Kazanskaya, O. *et al.* R-Spondin2 is a secreted activator of Wnt/beta-catenin signaling and is required for Xenopus myogenesis. *Developmental cell* **7**, 525-534, doi:10.1016/j.devcel.2004.07.019 (2004).
17 Maguschak, K. A. & Ressler, K. J. Beta-catenin is required for memory consolidation. *Nature neuroscience* **11**, 1319-1326, doi:10.1038/nn.2198 (2008).
18 Ferraro, L. *et al.* Neurotensin receptors as modulators of glutamatergic transmission. *Brain research reviews* **58**, 365-373, doi:10.1016/j.brainresrev.2007.11.001 (2008).
